# Altitude modulates growth and bioactive compounds in two *Gastrodia elata* forms through the microenvironment and soil microbes

**DOI:** 10.3389/fpls.2026.1734174

**Published:** 2026-03-05

**Authors:** Haixin Diao, Daichuan Pan, Junfei Wang, Shunxing Guo

**Affiliations:** 1 Institute of Medicinal Plant Development, Chinese Academy of Medical Sciences and Peking Union Medical College, Beijing, China; 2 Biotechnology Institute of Guizhou, Guizhou Academy of Agricultural Sciences, Guiyang, China

**Keywords:** altitude, bioactive compounds, culturable bacteria, developmental stage, *Gastrodia elata* forms, soil enzyme activity, structural equation modeling

## Abstract

**Introduction:**

*Gastrodia elata* Bl. is a medicinal-edible heterotrophic orchid with distinct vertical distribution, but unstable yield and inconsistent quality in cultivation limit its industrial development. The mechanisms by which altitude modulates growth and bioactive compound accumulation in different *G. elata* forms remain unclear.

**Methods:**

We conducted a two-factor field experiment (two forms: *G. elata* f. *glauca*, *G. elata* f. *elata*; three altitudes: 650, 1653, 1953 m) in the Qinba Mountains, using a consistent commercial *Armillaria sp*. strain to isolate bacterial effects. We analyzed microclimate, soil properties, soil enzyme activities, culturable bacterial communities, and tuber bioactive compounds (gastrodin, parishins) across developmental stages.

**Results:**

Form-specific altitudinal responses were observed: total yield peaked at high altitude (1953 m; 2668.11 ± 317.10 g), while bioactive compounds were enriched at middle altitude (1653 m)—with optimal accumulation at the Large *Baima* stage (f. *glauca*) and *Mima* stage (f. *elata*). Soil pH was the primary correlative factor for f. *glauca* quality (explaining 52%–70.5% of variation in RDAs), whereas integrated carbon-acquiring enzyme activity (16.6%) was key for f. *elata*—consistent with PLS-SEM evidence of an indirect “soil properties→enzyme activities” pathway for f. *elata* quality. Culturable tuber-associated bacteria (dominated by Pseudomonadota, least diverse at middle altitude) correlated divergently with yield and quality: positively with yield but negatively with quality in f. *glauca*; weakly positive with yield and strongly (non-significantly) positive with quality in f. *elata* (*p* < 0.05).

**Discussion:**

Our findings clarify form-specific correlative networks linking altitude, microenvironment, soil microbes, and plant performance, providing targeted guidance for ecological cultivation to balance high yield and quality in *G. elata*.

## 1 Introduction

*Gastrodia elata* Bl. (Orchidaceae) is a heterotrophic orchid whose tubers serve as both food and medicinal material (Yuan et al., 2018; Wang, 2020). These tubers accumulate bioactive compounds—primarily gastrodin and parishins—linked to neurological benefits (Yu et al., 2022). Two main forms are cultivated: *G. elata* f. *elata*—widely planted and higher-yielding—and *G. elata* f. *glauca*, which is less widely cultivated due to lower yields (Zhang and Wang, 2022). Comparative analyses indicate f. *glauca* tubers often contain higher concentrations of bioactive compounds (Xue et al., 2022; Wang et al., 2022), suggesting a potential yield-quality trade-off at the form level. In practice, achieving both high yield and consistent quality remains challenging, with variability likely driven by planting material, symbiotic fungi, and environmental conditions (Yang et al., 2021; Wang C. H. et al., 2023; Wang H. J. et al., 2023).

Ecologically, the two forms exhibit divergent distributions: f. *glauca* occurs more frequently at higher altitudes, while f. *elata* dominates lower altitudes (Xu, 1993)—a pattern suggesting adaptive differences in growth and metabolism. Altitude shapes local microclimate and soil properties (Chen et al., 2022), thereby influencing plant physiology. For example, cultivating f. *elata* at ~1800–2000 m is associated with improved tuber quality (Li et al., 2024), and gastrodin accumulation varies across developmental stages (Zhu et al., 2024). Recent multi-factor studies highlight plant form, *Armillaria* relative abundance, soil available potassium, and temperature seasonality as key variables (Jiang et al., 2025). A critical gap persists: how these variables—particularly altitude, form, and developmental stage—interact under field conditions to shape growth and chemical composition.

Tuber-associated microbial communities also play important roles. As a heterotrophic plant, *G. elata* depends on symbiotic fungi for nutrition (Xu, 1993; Guo et al., 1999; Wang et al., 2020), and its tuberosphere harbors diverse bacterial assemblages (Liu, 2022). To isolate bacterial contributions independently of fungal symbionts, we utilized a single consistent commercial *Armillaria* strain for both forms—focusing on culturable bacterial assemblages in relation to altitude, form, and developmental stage. These bacteria are environmentally sensitive, with composition linked to soil nutrient cycling (Wang et al., 2021); notably, for instance, *Rahnella* spp. from the *G. elata* tuberosphere promote *Armillaria* growth (Liu et al., 2022), directly linking bacterial activity to the plant’s symbiotic nutrition system. Nevertheless, a systematic understanding of how culturable bacterial assemblages vary with altitude, form, and developmental stage remains lacking.

We tested four hypotheses: (1) Abiotic conditions (microclimate and soil properties) and soil enzyme activities may differ with altitude and form. (2) Tuber growth and bioactive compound accumulation may respond non-linearly to altitude, with form-specific differences across developmental stages. (3) Culturable tuber-associated bacterial diversity and composition may vary with altitude, form, and developmental stage. (4) Plant performance may correlate with an integrated network linking abiotic conditions, soil enzymes, and culturable bacterial traits.

This study provides a multi-altitude field comparison of the two main *G. elata* forms. By monitoring microclimate, soil properties, and analyzing stage-specific growth traits, bioactive compounds, soil enzymes, and culturable microbes, we clarify key correlative relationships within the “environment–microbe–plant” system. Our findings advance understanding of the correlative drivers underlying yield and quality, offering practical insights for cultivation management.

## 2 Materials and methods

### 2.1 Experimental design and site description

Fieldwork was conducted in the Qinba Mountain area of Sichuan Province, China (**Supplementary Table 2**). We employed a two-factor factorial design: two *G. elata* forms (f. *glauca*, f. *elata*) and three altitudes (650, 1653, 1953 m). For each form-altitude combination, five replicate cultivation beds (1.6 m × 0.65 m × 0.1 m) were established. Seed tubers of *G. elata* f. *glauca* were sourced from Qingchuan, Sichuan; *G. elata* f. *elata* from Hanzhong, Shaanxi. The symbiotic fungus was a commercial *Armillaria* sp. strain (designated as Ar6, provided by Chunguang Fungiculture Development Co., Ltd.), and “Ar6 spawn” (used in cultivation) is equivalent to this Ar6 *Armillaria* sp. strain—specifically, third-generation solid spawn cultured with small *Cyclobalanopsis glauca* wood sticks (5 cm × 2 cm), a standard practice for *G. elata* cultivation (Xu, 1993). All beds were planted simultaneously (April 28–May 1, 2022) with 500 g seed tubers, 10 bottles of Ar6 spawn (500 g/bottle), and 45 *Cyclobalanopsis glauca* wood segments (15 cm × 7 cm). Tubers were harvested between March 15 and 17, 2023, across the three altitudes. Sampling was conducted within 3 consecutive days to ensure consistency across altitudes, with no significant environmental fluctuations during this period.

One automated weather station (HW-D4, China) was installed at each altitude (**Supplementary Figure 1**). Continuous monitoring began on July 1, 2022 (due to procurement delays) and ended March 31, 2023 (273 days), following GB/T35221-2017. Air temperature (AT), relative humidity (RH), 10 cm soil temperature (ST), soil moisture (SM), and soil electrical conductivity (SEC) were recorded at 30-minute intervals; daily averages were used for analysis.

### 2.2 Sample collection and processing

At harvest, tubers from each cultivation bed were classified into seven developmental stages based on morphology and weight: Large, Medium, and Small *Jianma* (mature tubers with flower buds); Large, Medium, and Small *Baima* (immature tubers); and *Mima* (juvenile tubers < 2 cm; **Table 1**). For each stage, 30 longest tubers per bed were measured for length (cm), width (cm), and individual weight (g); total yield (g) and tuber count per stage and per bed were recorded. For culturable microbial and enzymatic analyses, three focal stages were prioritized: Medium *Jianma* (MJ), Large *Baima* (LB), *Mima* (M)—covering principal maturation phases, selected based on the following: both forms have MJ (a consistent representative of fully mature tuber); Large *Jianma* (LJ) was excluded due to its absence in f. glauca at middle altitude; Small *Jianma* (SJ) was avoided as it is the early transitional stage from LB (immature) to fully mature tuber, which would reduce differentiation between *Baima* and *Jianma* phases and obscure their distinct functional differences.

**Table 1:** Classification of tubers from two *G. elata* forms into seven developmental stages based on morphology and weight.

Developmental stages	Specifications	Length (cm)	Width (cm)	Individual weight (g)	Morphological characteristics
Large J*ianma*	Large J*ianma*-type Mature tuber	> 9	>3.5	> 80	mature tuber with red flower stem buds
Medium *Jianma*	Medium *Jianma*-type Mature tuber	6 - 9	2 - 3.5	30 - 80	
Small *Jianma*	Small *Jianma*-type Mature tuber	2 - 8	1 - 3	10 - 40	
Large *Baima*	Large *Baima*-type Immature tuber	> 6	> 2	5 - 30	immature tuber with snow-white head-like buds
Medium *Baima*	Medium *Baima*-type Immature tuber	4 - 6	1 - 2	6 - 10	
Small *Baima*	Small *Baima*-type Immature tuber	2 - 5	0 - 1.5	< 6	
*Mima*	*Mima*-type Juvenile tuber	< 2	–	–	Juvenile tuber is less than 2 cm in length immature tuber

Length and Width refer to the longest and widest dimensions (cm) of the tuber, respectively. Note that for the *Mima* stage, Width and individual weight were not quantified due to small tuber size.

Two soil compartments were sampled (three beds randomly selected per altitude). Tuberosphere soil (within 1 mm of tuber surfaces) was collected from the three stages (Edwards et al., 2015; Kuzyakov and Razavi, 2019); soil from the same stage within a bed was composited (n = 1 per bed; total n = 3 per altitude). Bulk soil (5–20 cm depth, > 1 mm from tubers) was sampled in an “S” pattern, with five subsamples composited per bed (Liu, 2022). All samples were transported on ice. Upon arrival, each soil sample was first air-dried: both tuberosphere soil and bulk soil were used for enzymatic assays after air-drying. Additionally, a separate portion of tuberosphere soil (not air-dried) was stored at -80 °C exclusively for microbial analysis, while bulk soil was further processed for physicochemical assays following air-drying.

### 2.3 Soil physicochemical and enzymatic analyses

Soil pH was measured in a 1:2.5 (soil:water, w/v) suspension. Exchangeable calcium (ECa) and magnesium (EMg) were determined via ammonium acetate extraction-atomic absorption spectrometry. Available micronutrients (AFe, AMn, ACu, AZn) were extracted with diethylenetriaminepentaacetic acid (DTPA; Lindsay and Norvell, 1978). Soil texture was analyzed via the pipette method (Lv and Li, 2010). Alkali-hydrolyzable nitrogen (AN) was measured by alkaline hydrolysis diffusion. Available potassium (AK) and phosphorus (AP) were quantified by inductively coupled plasma optical emission spectrometry (ICP-OES). Soil organic carbon (SOC) was determined using the Walkley-Black method (Walkley and Black, 1934; Bao, 2000). All procedures followed Chinese agricultural and forestry standards (**Supplementary Table 3**).

To assess microbial nutrient acquisition potential, six extracellular enzyme activities were measured using commercial kits (Solarbio, China; kit details: **Supplementary Table 4**). β-glucosidase (BG; EC 3.2.1.21), β-1,4-cellobiohydrolase (CBH; EC 3.2.1.91), and β-xylosidase (XS; EC 3.2.1.37) activities were summed as “Enzyme C” (carbon acquisition). β-N-acetylglucosaminidase (NAG; EC 3.2.1.52) and leucine aminopeptidase (LAP; EC 3.4.11.1) activities were summed as “Enzyme N” (nitrogen acquisition) (Sinsabaugh et al., 2010; Peng and Wang, 2016). Acid phosphatase activity (Enzyme P; EC 3.1.3.2) represented phosphorus acquisition.

### 2.4 Analysis of tuber bioactive compounds

Fresh tubers were steamed in a steamer over boiling water (water-separated steaming, traditional Chinese Paozhi) to inactivate enzymes (duration adjusted by stage: 30 min for LJ, 25 for MJ, 20 for SJ, 15 for LB, 10 for MB, 8 for SB, 5 for M) until translucent (Chinese Pharmacopoeia Commission, 2025). Steamed tubers were cooled, sliced, dried at 60 °C to constant weight, ground into powder, and passed through a No. 4 sieve. Reference standards (gastrodin [GA], *p*-hydroxybenzyl alcohol [HBA], parishins A/B/C/E; **Supplementary Table 4**) were prepared as 0.4 mg·mL^-^¹ stock solutions in acetonitrile-water (3:97, v/v). For analysis (Liu et al., 2019), 2.0 g powder was extracted with 20 mL 50% aqueous methanol via sonication (250 W, 60 min, room temperature). After cooling, weight compensation, and filtration, extracts were diluted with acetonitrile-water and analyzed by UPLC using an ACQUITY UPLC HSS T3 column (2.1 × 100 mm, 1.8 μm) at 40 °C. Mobile phase: (A) acetonitrile, (B) 0.05% phosphoric acid; gradient elution (0–8.5 min: 3% A; 8.5–9 min: 3–12% A; 9–13 min: 12–18% A) at 0.300 mL·min^-^¹ (Chen et al., 2019). Detection was at 220 nm with a 5 μL injection volume (Chen et al., 2019; Liu et al., 2019). Three independent extracts per sample were prepared, each injected in duplicate.

The UPLC method was validated: linear calibration curves (0.005-0.4 mg/mL, *R*² > 0.999), 48-hour sample stability (RSD < 2%), instrumental precision (six consecutive standard injections), method repeatability (six independent extracts), and recovery (50%, 100%, 150% spiking levels) all met acceptable standards (**Supplementary Table 5**).

### 2.5 Isolation and analysis of culturable tuber-associated microorganisms

A culture-dependent approach was used to isolate culturable tuber-associated microbiota (Hafeez et al., 2006; Zhang, 2013; Liu and Yao, 2022)—results thus reflect this subset, not the complete *in situ* community. For tuberosphere soil: 3 g soil was suspended in sterile saline, shaken, centrifuged to obtain a 10^-^¹ dilution (A). Tubers were ultrasonicated in saline, and the suspension diluted to 10^-^¹ (B). Equal volumes of A and B were mixed to create a 10^-^¹ working dilution. For endophytes: ultrasonicated tubers were surface-sterilized (75% ethanol + 5% sodium hypochlorite), homogenized in saline, and diluted to 10^-^¹. Serial dilutions (10^-^² to 10^-6^) were prepared; 50 μL of 10^-4^ and 10^-6^ dilutions were spread in triplicate on LB agar (bacteria) and PDA (fungi). Plates were incubated at 12 °C, 16 °C, 20 °C for 5–7 days. Morphologically distinct colonies/hyphae were subcultured to obtain pure isolates.

Genomic DNA was extracted from 466 culturable bacterial isolates using the ThermoFisher Platinum™ Direct PCR Universal Master Mix (USA) and from 7 culturable fungal isolates using the HerBOL Plant/Animal/Fungi/Chinese Medicine DNA Extraction Kit (China). For bacteria, the V3-V4 region of 16S rRNA was amplified with primers 515F (5′-GTGCCAGCMGCCGCGGTAA-3′) and 806R (5′-GGACTACHVGGGTWTCTAAT-3′) (Zhao et al., 2022): 94 °C for 3 min; 40 cycles (94 °C 30 s, 52 °C 30 s, 72 °C 30 s); 72 °C for 5 min. For fungi, the ITS region was amplified with primers ITS1 (5′-TCCGTAGGTGAACCTGCGG-3′) and ITS4 (5′-TCCTCCGCTTATTGATATGC-3′) (White et al., 1990): 94 °C for 3 min; 35 cycles (94 °C 30 s, 55 °C 30 s, 72 °C 1 min); 72 °C for 5 min. Amplicons were sequenced at the Major Platform Service Center of the Institute of Crop Sciences, Chinese Academy of Agricultural Sciences. Sequences were quality-filtered, clustered into OTUs (≥ 99% similarity) using CodonCode Aligner 11.0.1, and taxonomically assigned against the NCBI 16S database. Culturable bacterial alpha diversity (Shannon-Wiener [H’], Simpson [SI], evenness [E]) was calculated based on OTU relative abundance (P_i_ = n_i_/N): H’ = -∑(P_i_lnP_i_); SI = 1 - ∑(P_i_)²; E = H’/lnS (S = total OTUs, n_i_ = individuals in OTU i, N = total individuals; Sarma et al., 2018; Diao et al., 2020; Qiu et al., 2024).

### 2.6 Statistical analysis

All analyses used α = 0.05. Data management was performed in Microsoft Excel 2019. Primary analyses (Linear Mixed Models [LMMs], Generalized Linear Mixed Models [GLMMs], General Linear Models [GLMs]) were conducted in IBM SPSS v26.0. Multivariate analyses (Redundancy Analysis [RDA], Partial Least Squares Structural Equation Modeling [PLS-SEM]) were implemented in Canoco 5 and R v4.5.1 (plspm package), respectively. Additional analyses (PCA, Mantel tests, correlation heatmaps) were run using ChiPlot (https://www.chiplot.online), and Venn diagrams were generated with Jvenn (http://jvenn.toulouse.inra.fr/app/example.html). Figures were created in GraphPad Prism 9 and assembled in Adobe Illustrator 2024 and Adobe Photoshop CC 2019.

Model selection was guided by data structure: (1) Stage-level variables (e.g., enzyme activities, tuber morphology): LMMs/GLMMs with altitude, form, developmental stage, and their interactions as fixed effects, and cultivation bed as a random intercept; GLMMs with a negative binomial distribution were applied to tuber count. (2) Bed-level variables (e.g., bulk soil properties, total yield): GLMs with altitude and form as fixed factors. Model assumptions (normality, homoscedasticity) were validated via diagnostic plots (Q-Q plots, residual vs. fitted plots) and Levene’s test (**Supplementary Figure 3–4**; **Supplementary Tables 6–8, 10-13**). *Post-hoc* pairwise comparisons were carried out using the Šidák test. Separate PLS-SEM models were constructed for each form, with variables selected *a priori* based on three criteria: (1) biological relevance (supported by literature linking factors like AK, pH, and enzyme activities to *G. elata* growth; Jiang et al., 2025); (2) significant correlations with dependent variables (growth traits/bioactive compounds) identified via RDA, Mantel tests, and correlation heatmaps; (3) no severe multicollinearity (Variance inflation factor [VIF] < 3). Reflective measurement models and estimation details are provided in **Supplementary Table 9**. The models were validated via bootstrapping with 1,000 resamples, which confirmed convergent validity (average variance extracted [AVE] > 0.5), and demonstrated satisfactory goodness-of-fit (GoF) indices (**Supplementary Figure 5**).

## 3 Results

### 3.1 Variation in soil properties and enzyme activities with altitude and *G. elata* form

#### 3.1.1 Microclimatic variation across altitudes

**Figure 1:**
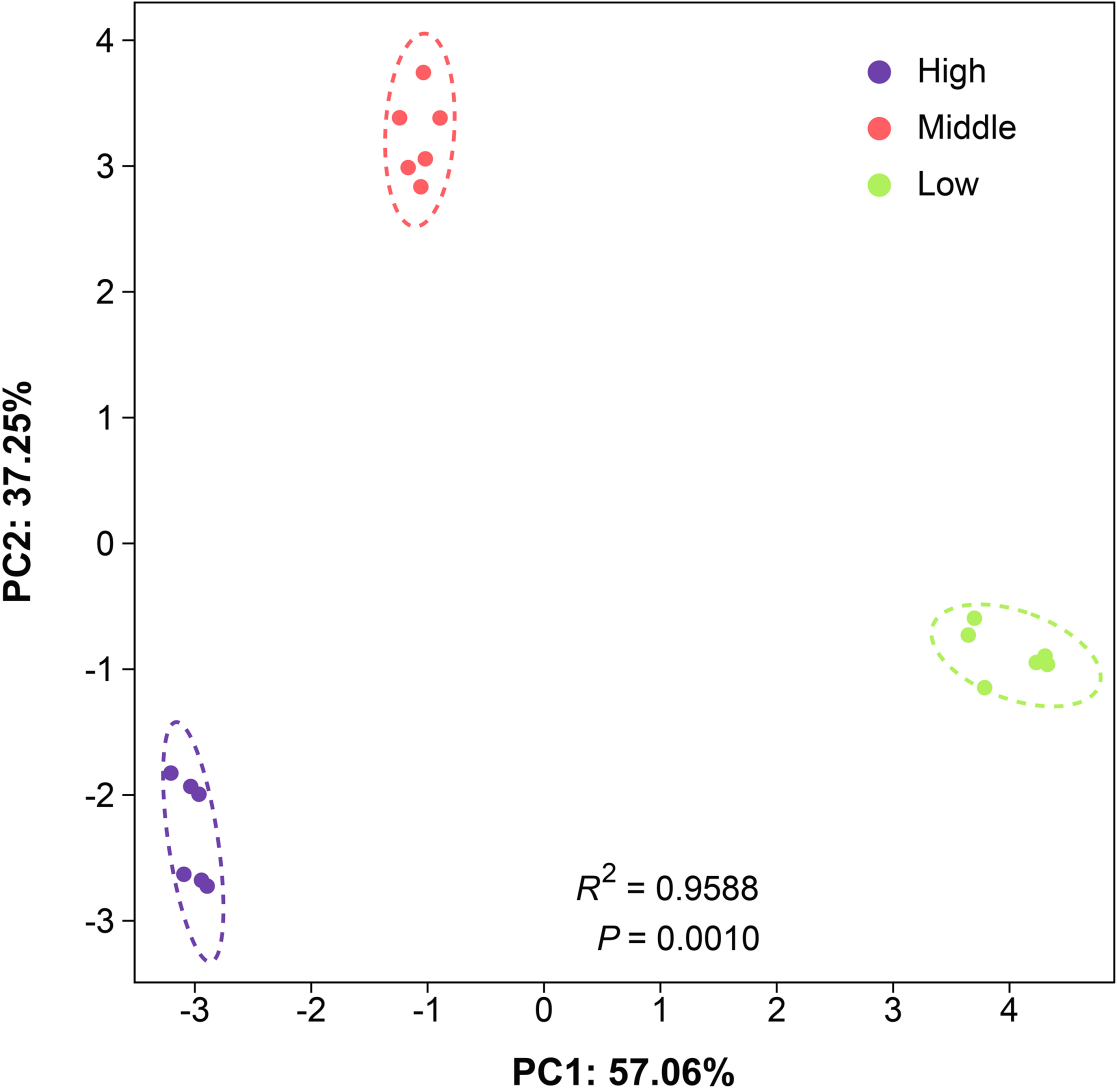
PCA of environmental factors across altitudes (n = 3 per altitude).

During cultivation (July-March), microclimate and soil properties varied markedly with altitude (**Supplementary Table 2**). Mean ST and AT decreased with elevation: high-altitude ST averaged 8.97 ± 7.44 °C (reflecting seasonal variation). SM and SEC increased with altitude (35.97 ± 8.49% and 190.93 ± 30.9 μs·cm^-^¹, respectively). PCA of combined microclimate and soil data showed clear separation by altitude (*R*² = 0.959, *p* = 0.001; **Figure 1**), confirming distinct environmental conditions across sites.

#### 3.1.2 Bulk soil physicochemical properties

**Figure 2:**
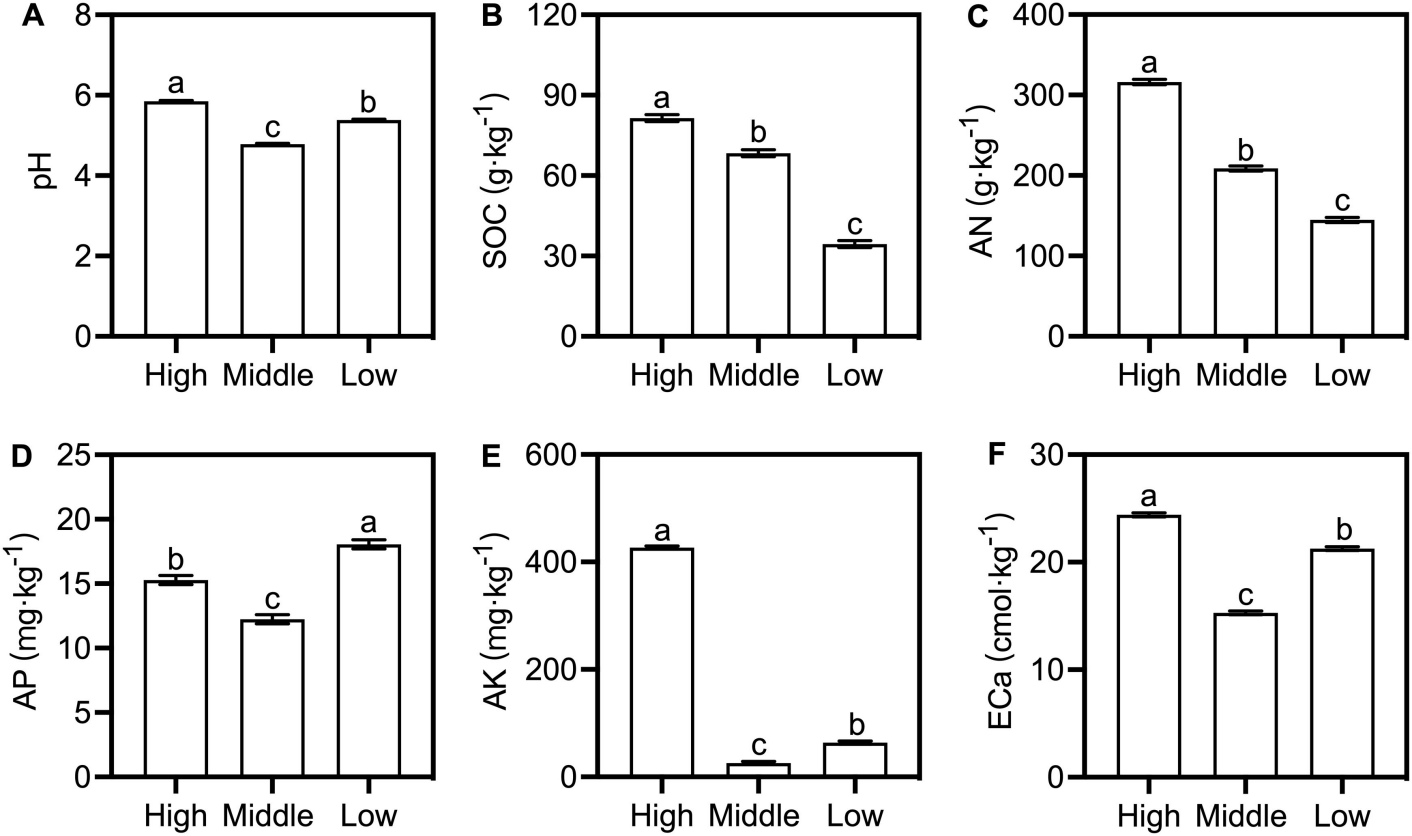
Bulk soil physicochemical properties across altitudes for two *G. elata* forms. Bars show mean ± standard error (SE; n = 3 cultivation beds). **(A)** pH; **(B)** SOC; **(C)** AN; **(D)** AP; **(E)** AK; **(F)** ECa. Different uppercase (f. *glauca*) and lowercase (f. *elata*) letters denote significant differences across altitudes; asterisks indicate significant differences between forms at a specific altitude (Šidák's test: ^*^p < 0.05, ^**^p < 0.01, ^***^p < 0.001). Abbreviations are defined in **Supplementary Table 1**.

Soil properties varied profoundly along the altitudinal gradient, with subtle modulation by form. GLMs identified altitude as the predominant explanatory factor (explaining 92%-100% of variance, all *p* < 0.001; **Supplementary Table 10**). pH, SOC, AN, AK, and ECa were elevated at high altitude (**Figure 2**). pH ranged narrowly (4.78–5.85, acidic), while nutrient concentrations varied dramatically: SOC, AN, and ECa at the high altitude were 1.60–2.36-fold higher than at the low altitude and 1.15–1.51-fold higher than at the middle altitude. AK showed the most pronounced altitudinal variation: high-altitude concentrations (426.74 ± 2.97 mg·kg^-^¹) exceeded middle and low sites by 16.43 and 6.71-fold. AP was inversely related to altitude (peak at low altitude: 12.24-18.05 mg·kg^-^¹). Form and altitude × form interactions were significant for most properties (**Supplementary Table 10**). For example, at middle altitude, f. *glauca* soils had higher pH, SOC, and AK than f. *elata* (**Supplementary Figure 6**); at high/low altitudes, this reversed for SOC, AN, AK, and ECa. AP was consistently higher in f. *glauca* soils across all altitudes.

#### 3.1.3 Tuberosphere soil enzyme activities

**Figure 3:**
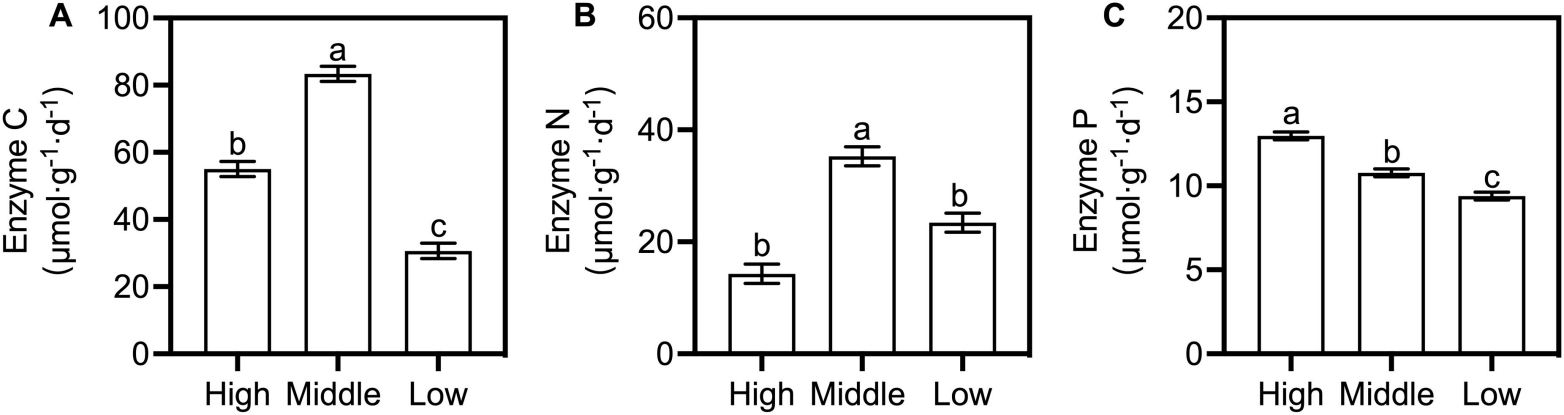
Tuberosphere soil enzyme activities across altitudes for two *G. elata* forms. Bars show mean ± SE (n = 3 cultivation beds). **(A)** Enzyme C; **(B)** Enzyme N; **(C)** Enzyme P. Different uppercase (f. *glauca*) and lowercase (f. *elata*) letters denote significant differences across altitudes; asterisks indicate significant differences between forms at a specific altitude (Šidák's test: ^*^p < 0.05, ^**^p < 0.01, ^***^p < 0.001). Abbreviations are defined in **Supplementary Table 1**.

Enzyme C (C acquisition), Enzyme N (N acquisition), and Enzyme P (P acquisition) showed distinct variation with altitude, form, and developmental stage. LMMs confirmed altitude as the strongest explanatory factor (Enzyme C: *F* = 141.3, *p* < 0.001; Enzyme N: *F* = 35.0, *p* < 0.001; Enzyme P: *F* = 49.7, *p* < 0.001; **Supplementary Table 11**). Middle altitude supported the highest Enzyme C (83.37 ± 2.27 μmol·d^-^¹·g^-^¹) and Enzyme N (35.26 ± 1.71 μmol·d^-^¹·g^-^¹)—1.52-2.72-fold and 1.50-2.47-fold higher than high/low altitudes, respectively. Enzyme P increased with elevation (9.39 ± 0.24 μmol·d^-^¹·g^-^¹ at low altitude to 12.98 ± 0.24 μmol·d^-^¹·g^-^¹ at high altitude; **Figure 3**).

Developmental stage was significant for Enzyme C (*F* = 27.6, *p* < 0.001). Altitude × form interactions were significant for all enzymes (**Supplementary Table 11**), with form-related differences shifting across altitudes (**Supplementary Figure 7**). A three-way interaction (altitude × form × stage) for Enzyme N (*F* = 5.3, *p* = 0.011) indicated stage-dependent altitudinal variation.

#### 3.1.4 RDA of soil properties and enzymes

**Figure 4:**
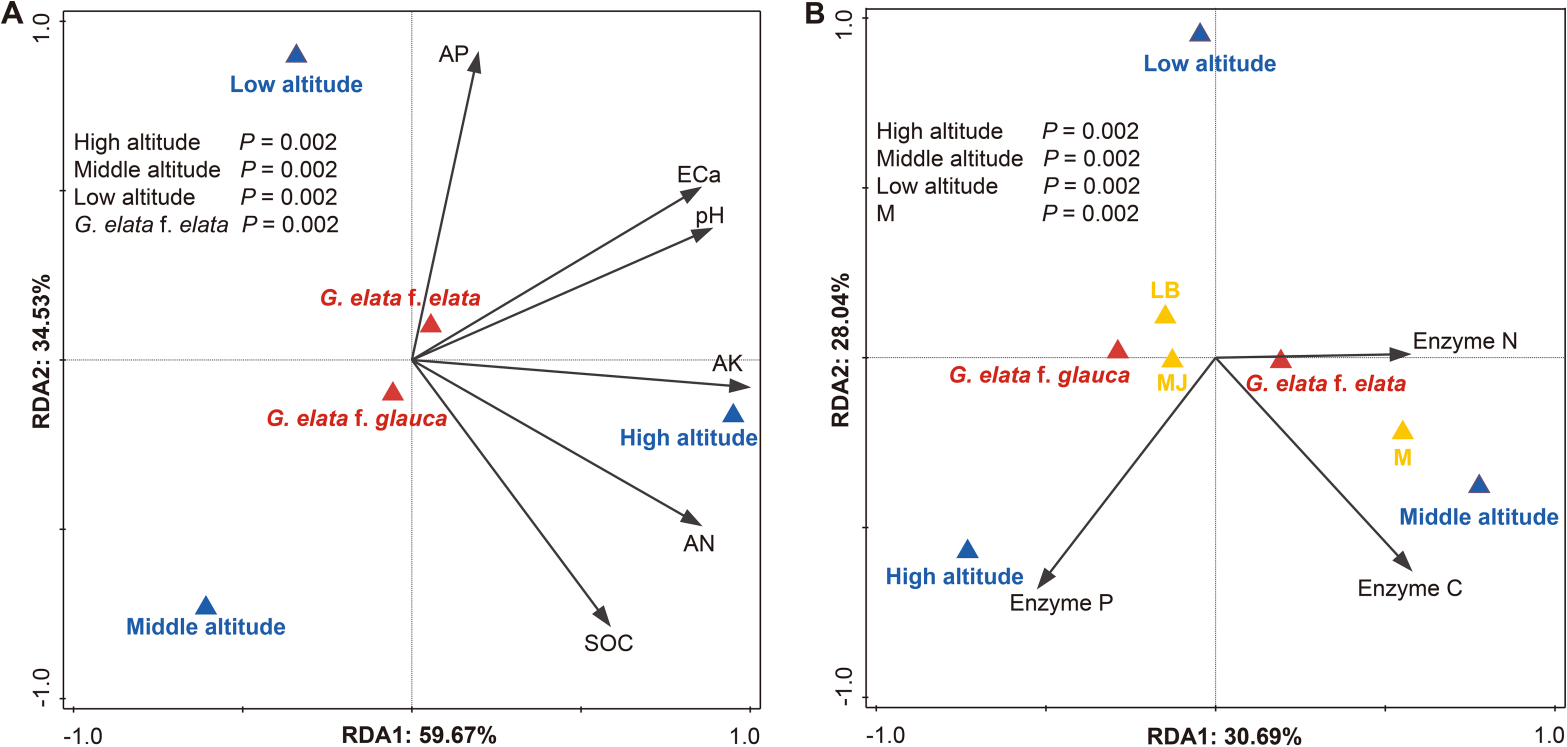
RDA of soil variables across altitude and plant factors (n = 3 cultivation beds). **(A)** Bulk soil physicochemical properties in relation to altitude and *G. elata* form. **(B)** Tuberosphere soil enzyme activities in relation to altitude, form, and developmental stage. Soil variables are represented by black arrows; triangles denote categorical factors: altitude (blue: High, Middle, Low), form (red: f. *glauca*, f. *elata*), and developmental stage (yellow: MJ, LB, M). *p* < 0.05. Abbreviations are defined in **Supplementary Table 1**.

RDA confirmed altitude as the predominant explanatory variable. For bulk soil properties, altitude explained 58.8% of the constrained variation (pseudo-*F* = 22.8, *p* = 0.002), while form contributed 3.7% (*p* < 0.05; **Figure 4A**). For tuberosphere soil enzymes, altitude accounted for 25–27% of the constrained variation (all *p* = 0.002), with developmental stage contributing 7.1% and form exerting a negligible effect (**Figure 4B**). Collectively, these results indicate that altitude is primarily associated with overall variation, while form and developmental stage are linked to context-dependent differences.

### 3.2 Variation in tuber growth traits with altitude and *G. elata* form

#### 3.2.1 Total tuber yield and count

**Figure 5:**
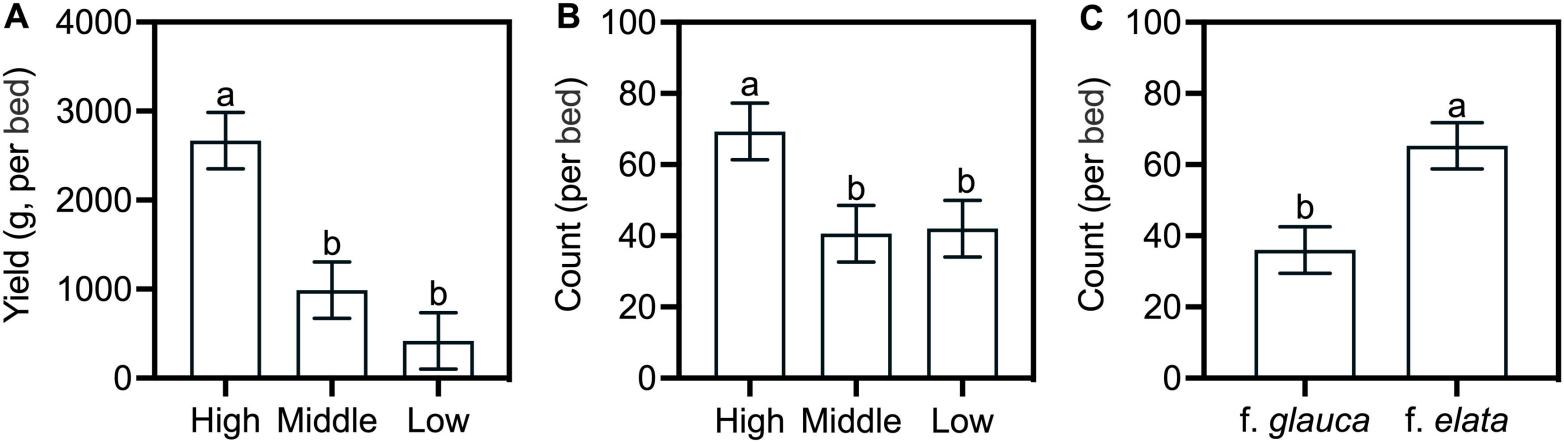
Total tuber yield and count across altitudes for two *G. elata* forms. Data are means ± SE (n = 5 cultivation beds). **(A)** Tuber yield (per bed); **(B)** Tuber count across altitudes (per bed); **(C)** Tuber count across forms (per bed). Different lowercase letters denote significant differences among altitudes (Šidák's test, *p* < 0.05). Tuber count represents the combined total from the *Jianma* and *Baima* stages per cultivation bed.

Bed-level analysis revealed a clear altitudinal signal in yield: total yield increased with altitude (*F* = 13.6, *p* < 0.001, η²_p_ = 0.53), peaking at high altitude (2668.11 ± 317.10 g; **Figure 5A**; **Supplementary Table 12**). Form and altitude × form interactions were non-significant for yield. Tuber count showed nuanced patterns: altitude (*F* = 4.1, *p* = 0.029, η²_p_ = 0.26) and form (*F* = 10.1, *p* = 0.004, η²_p_ = 0.30) were significant. High altitude had the highest mean count (69 ± 8), and *G. elata* f. *elata* count was 1.81-fold greater than *G. elata* f. *glauca* (**Figures 5B, C**). No altitude × form interaction was detected. Enhanced yield links to higher altitude, while tuber number reflects both elevation and form.

#### 3.2.2 Dynamics of growth traits across developmental stages

**Figure 6:**
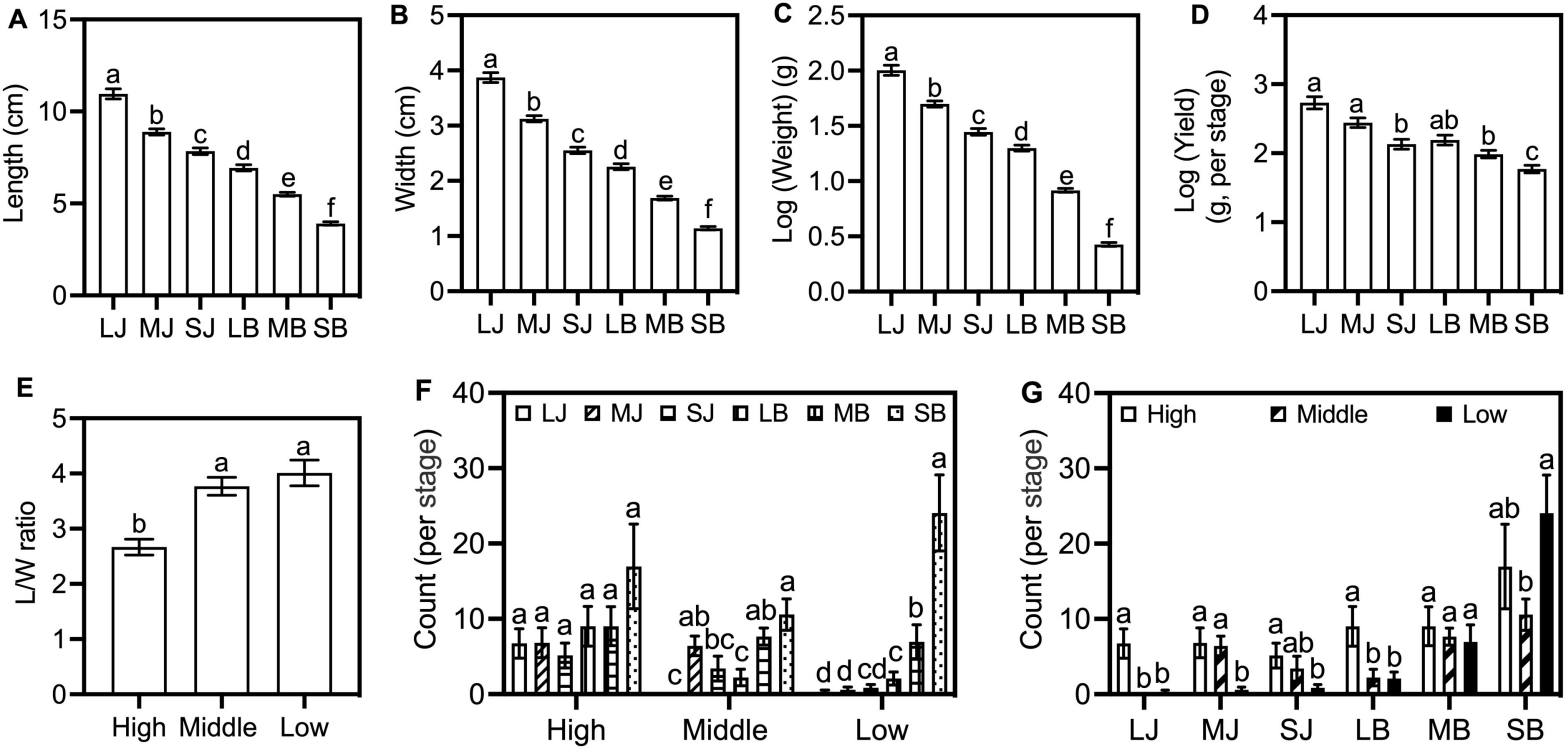
Variation in tuber growth traits across developmental stages and altitudes. **(A–D)** Trends in tuber length, width, log-transformed individual weight, and yield across six developmental stages (LJ to SB). **(E)** Tuber L/W ratio across three altitudes. **(F)** Tuber count across altitudes within each stage. **(G)** Tuber count across stages within each altitude. Data represent mean ± SE (n = 5 cultivation beds). Different lowercase letters indicate significant differences (Šidák's test, *p* < 0.05). Abbreviations are defined in **Supplementary Table 1**.

Mixed-effects models showed significant associations of altitude, form, and stage with growth traits (**Supplementary Table 13**). Stage had the strongest relationship—e.g., individual tuber weight (*F* = 948.0, *p* < 0.001). Tuber length, width, weight, and yield declined from Large *Jianma* to Small *Baima* (**Figures 6A–D**). Altitude linked to morphology (*F* = 16.4, *p* < 0.001): middle/low altitude L/W ratios were 1.4-1.5-fold higher than high altitude, indicating rounder tubers at high elevation (**Figure 6E**).

Tuber count was shaped by altitude × stage (*F* = 410.2, *p* < 0.001): at middle/low altitudes, counts varied across stages (peak at Small *Baima*); no stage variation was detected at high altitude. High altitude counts were generally highest, except Small *Baima* (max at low altitude: 24 ± 5; **Figures 6F, G**). Three-way interactions (altitude × form × stage) were significant for some traits but had smaller effect sizes (**Supplementary Figure 8**).

#### 3.2.3 RDA of growth traits

**Figure 7:**
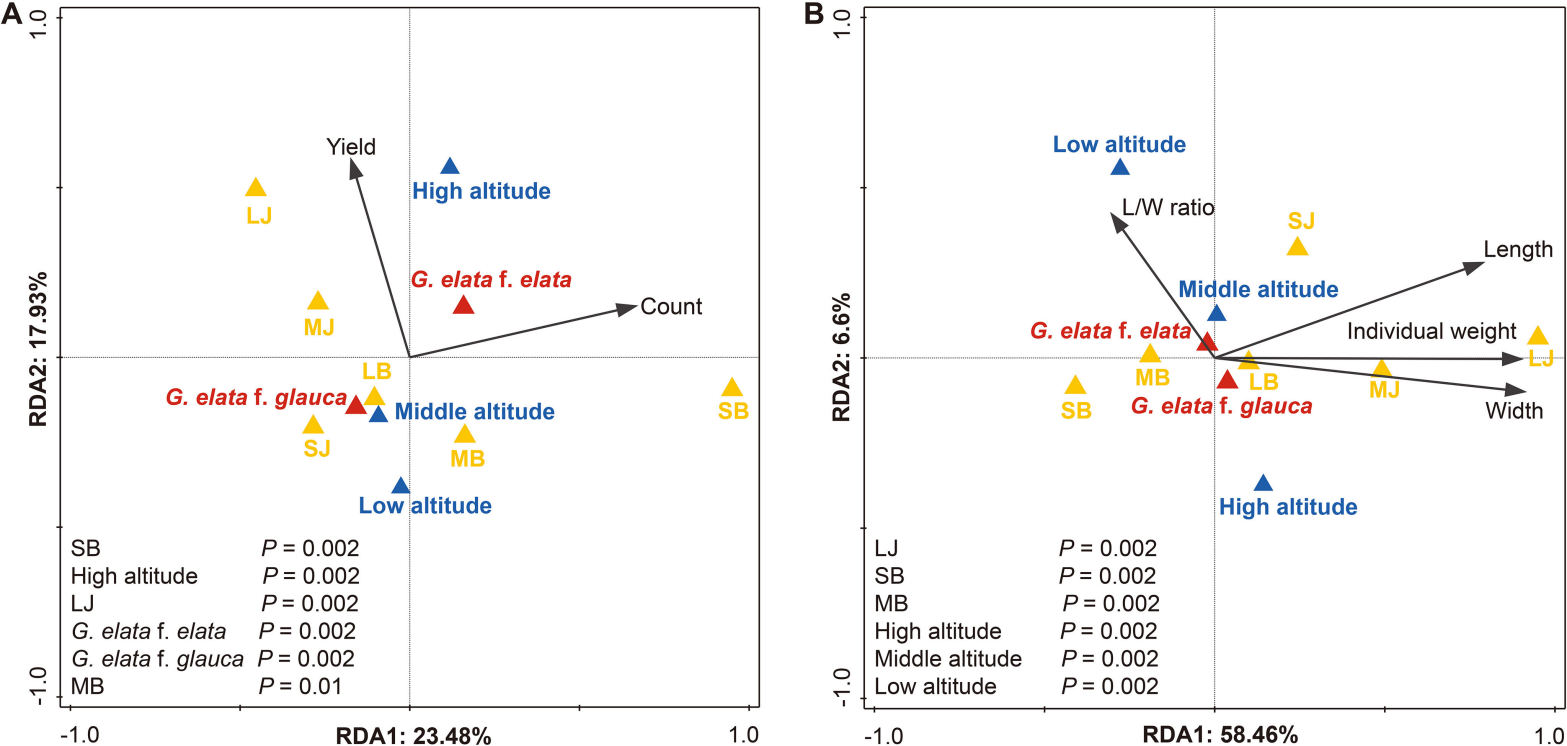
RDA of tuber growth traits in relation to altitude, form, and developmental stage (n = 5 cultivation beds). **(A)** Stage-specific yield and count. **(B)** Morphological traits (length, width, L/W ratio, individual weight). Black arrows represent growth traits; triangles show centroids for altitude (blue), form (red), and stage (yellow: LJ, MJ, SJ, LB, MB, SB). *p* < 0.05. Abbreviations are defined in **Supplementary Table 1**.

RDA for yield and count (per stage) explained 38.7% of adjusted variation, with the Small *Baima* stage and high altitude as key associated factors (**Figure 7A**)—aligning with the peak tuber count at Small *Baima* and high yield at high altitude. For tuber morphology (length, width, L/W ratio, weight), RDA explained 66.7% of variation: the Large *Jianma* stage contributed the most, followed by Small *Baima* stage (**Figure 7B**). Altitude showed consistent yet smaller associations with morphological traits, while form exerted minimal independent effects. Collectively, these results indicate that developmental stage is the primary factor associated with variation in tuber growth, with altitude linked to differences in yield and L/W ratio.

### 3.3 Variation in tuber bioactive compounds with altitude and *G. elata* form

**Figure 8:**
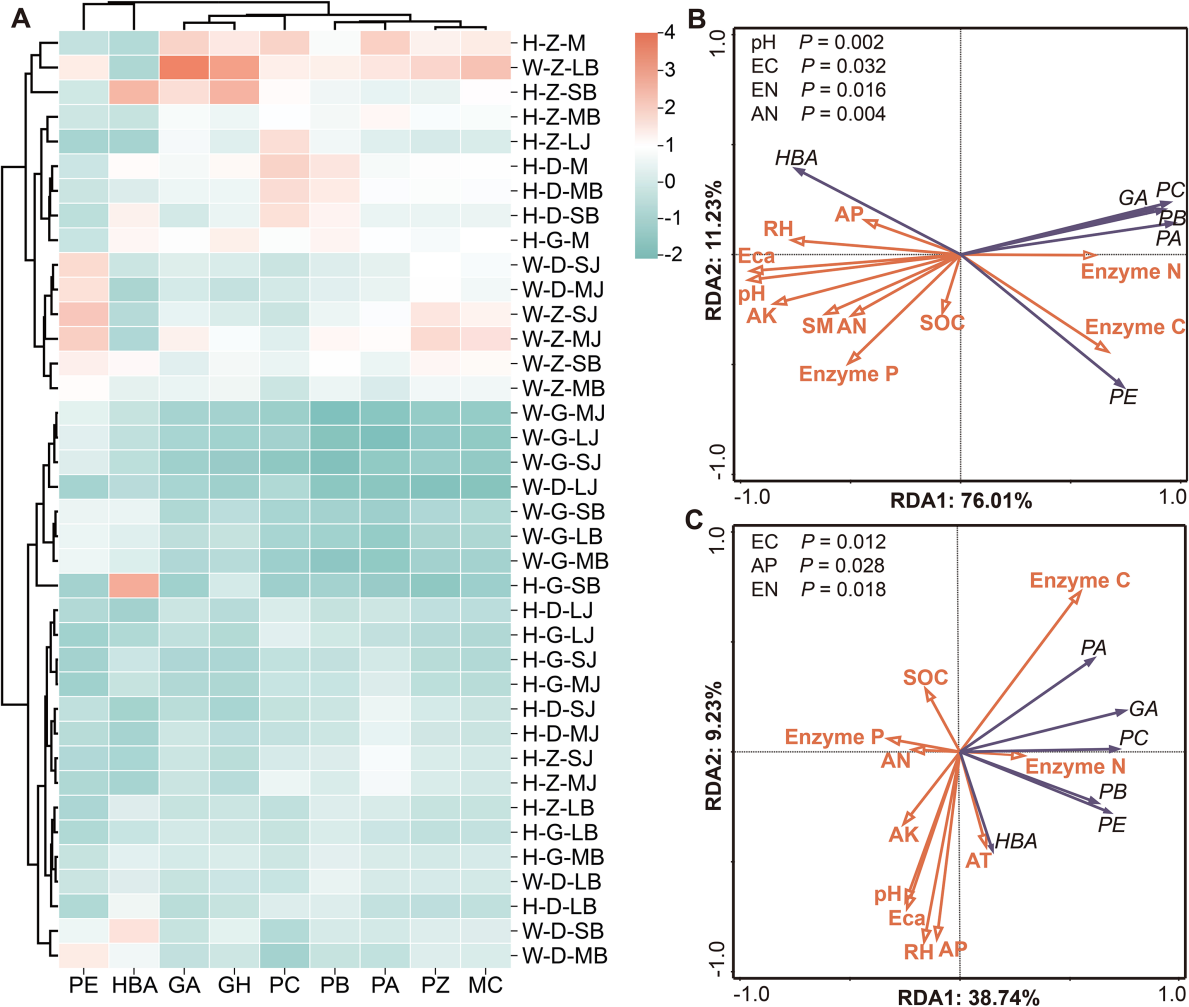
Composition and environmental associations of bioactive compounds in *G. elata* tubers. **(A)** Cluster heatmap of six bioactive compounds across samples (n = 3). **(B)** RDA ordination showing associations among microclimate, soil properties, soil enzyme activities, and bioactive compounds across three developmental stages (MJ, LB, M) of *G. elata f. glauca* (n = 8 samples). **(C)** Corresponding RDA for *G. elata* f. *elata* (n = 9 samples). Sample codes in **(A)**: Form (W, *G. elata* f. *glauca*; H, *G. elata* f. *elata*), Altitude (G, High; Z, Middle; D, Low), Stage (LJ, MJ, SJ, LB, MB, SB). Abbreviations are defined in **Supplementary Table 1**. *P* < 0.05.

Cluster analysis of six bioactive compounds revealed a clear altitudinal trend: concentrations were enriched at middle altitude, followed by low altitude, with the lowest at high altitude (**Figure 8A**). This pattern differed by form and stage: *G. elata* f. *glauca* GA (13.88 ± 0.18 mg·g^-^¹) and total bioactive compounds (126.59 ± 2.52 mg·g^-^¹) peaked at middle altitude Large *Baima*—3.33-fold and 1.72-fold higher than *G. elata* f. *elata* (**Supplementary Table 14**). *G. elata* f. *elata* GA (9.6 ± 0.04 mg·g^-^¹) and total compounds (108.73 ± 0.34 mg·g^-^¹) peaked at middle altitude *Mima* (**Supplementary Table 15**). Individual compounds showed specific trends: HBA was elevated in *G. elata* f. *elata* Small *Baima* at middle/high altitudes, while parishins exhibited distinct peak patterns—PA was highest in *G. elata* f. *elata Mima* at middle altitude, PB and PC peaked in *G. elata* f. *elata Mima* at low altitude, and PE was enriched in *G. elata* f. *glauca* Medium *Jianma* and Small *Jianma* at middle and low altitudes.

RDA explored links between microclimate, soil properties, enzymes, and bioactive compounds. For all seven stages, form-specific correlative factors emerged: variation in *G. elata* f. *glauca* bioactive compounds was primarily associated with pH (52%), while *G. elata* f. *elata* was linked to AK (16.7%; **Supplementary Figure 9A**). For the three focal stages (covering principal maturation phases), pH remained the most strongly associated factor for *G. elata* f. *glauca* (70.5%), with Enzyme C, Enzyme N, and AN also showing significant correlations (**Figure 8B**). For *G. elata* f. *elata*, Enzyme C/N and AP were the most prominent correlative factors, with pH playing a minimal role (**Figure 8C**). Form-specific environmental and biochemical contexts shape the accumulation of bioactive compounds.

### 3.4 Variation in culturable tuber-associated bacteria with altitude and *G. elata* form

#### 3.4.1 Diversity of culturable bacteria

A total of 473 microbial isolates were obtained: 7 culturable fungi and 466 culturable bacteria. The fungi (identified as *Coprinellus*, *Metarhizium*, *Ilyonectria*, *Aspergillus*, *Cordyceps*, *Sistotrema*; **Supplementary Table 16**)—not typical orchid symbionts. As these fungal sequences were not the focus of this study nor relevant to the core research question regarding bacterial communities, they were not deposited in a public database. The 466 bacteria clustered into 164 OTUs (**Table 2****;** focus of subsequent analysis). For both forms, tuberosphere OTU richness and Shannon indices were higher than endosphere (**Supplementary Figure 10**). Endophytes were rare and stage/altitude-dependent (**Supplementary Figure 11**).

**Table 2:** Alpha diversity indices of culturable tuber-associated bacterial assemblages.

Sample	*G. elata* f. *glauca*					*G. elata* f. *elata*				
	No. OTUs	No. isolates	H'	SI	E	No. OTUs	No. isolates	H'	SI	E
GJ-MJ	28	46	2.26	0.86	0.68	25	38	2.17	0.86	0.67
GJ-LB	15	26	1.93	0.84	0.71	8	18	1.91	0.84	0.92
GJ-M	13	20	1.84	0.79	0.72	22	37	2.02	0.83	0.65
ZJ-MJ	14	28	1.35	0.63	0.51	14	23	1.67	0.76	0.63
ZJ-LB	8	19	1.39	0.69	0.67	8	17	1.73	0.81	0.83
ZJ-M	–	–	–	–	–	0	0	–	1.00	–
DJ-MJ	13	23	2.10	0.86	0.82	14	20	2.17	0.87	0.82
DJ-LB	10	22	1.83	0.82	0.80	11	17	2.27	0.89	0.95
DJ-M	24	28	2.36	0.88	0.74	17	22	2.15	0.87	0.76
GN-MJ	0	0	–	1	–	0	0	–	1	–
GN-LB	0	0	–	1	–	1	1	0	0	0
GN-M	1	1	0	0	0	2	4	0	0	0
ZN-MJ	7	8	0.68	0.49	0.35	0	0	–	1	–
ZN-LB	2	7	0	0	0	0	0	–	1	–
ZN-M	–	–	–	–	–	0	0	–	1	–
DN-MJ	0	0	–	1	–	4	6	1.04	0.63	0.50
DN-LB	3	7	0.64	0.44	0.58	0	0	–	1	–
DN-M	6	14	1.56	0.78	0.87	6	12	1.01	0.61	0.56

Sample codes: altitude (G, high; Z, Middle; D, low), isolation source (J, tuberosphere soil; N, endosphere), developmental stage (MJ, Medium *Jianma*; LB, Large *Baima*; M, *Mima*). Diversity indices: H', Shannon-Wiener index; SI, Simpson’s index; E, Species evenness index. Isolates were not obtained for *G. elata* f. *glauca* at the middle-altitude *Mima* stage due to insufficient sample quantity.

**Figure 9:**
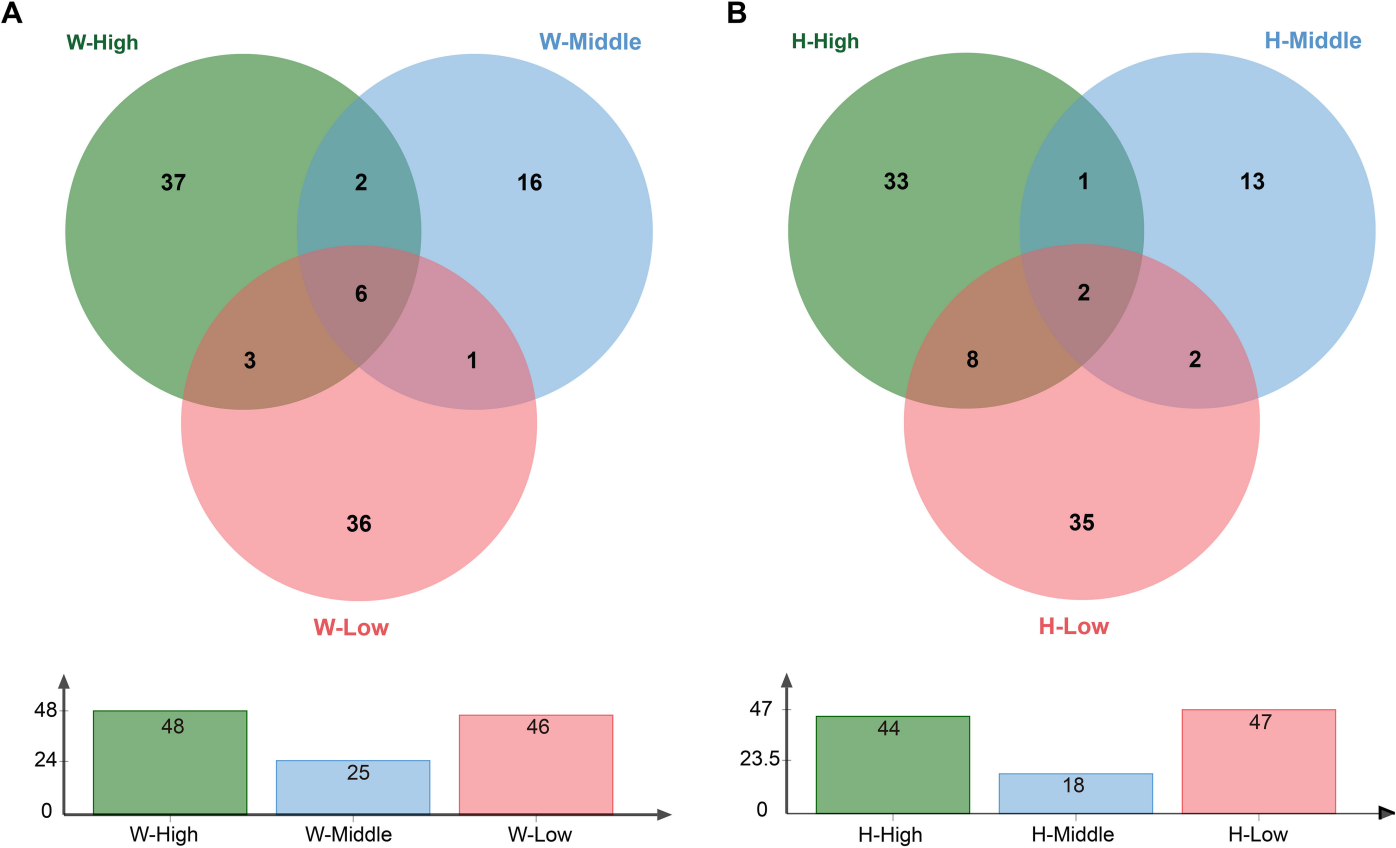
Venn diagrams of culturable tuber-associated bacterial OTUs across altitudes. **(A)** *G. elata* f. *glauca* (W; n = 8 samples). **(B)** *G. elata* f. *elata* (H; n = 9 samples).

Tuberosphere bacterial diversity was reduced at middle altitude for both forms (**Table 2**). Pooled OTU data (all stages) showed high/low altitudes had nearly double the OTUs of middle altitude (**Figure 9**). Unique OTUs were more abundant at high/low altitudes: *G. elata* f. *glauca* had 37/36 unique OTUs (high/low) vs. 16 (middle); *G. elata* f. *elata* had 33/35 (high/low) vs. 13 (middle).

#### 3.4.2 Composition of culturable bacterial assemblages

**Figure 10:**
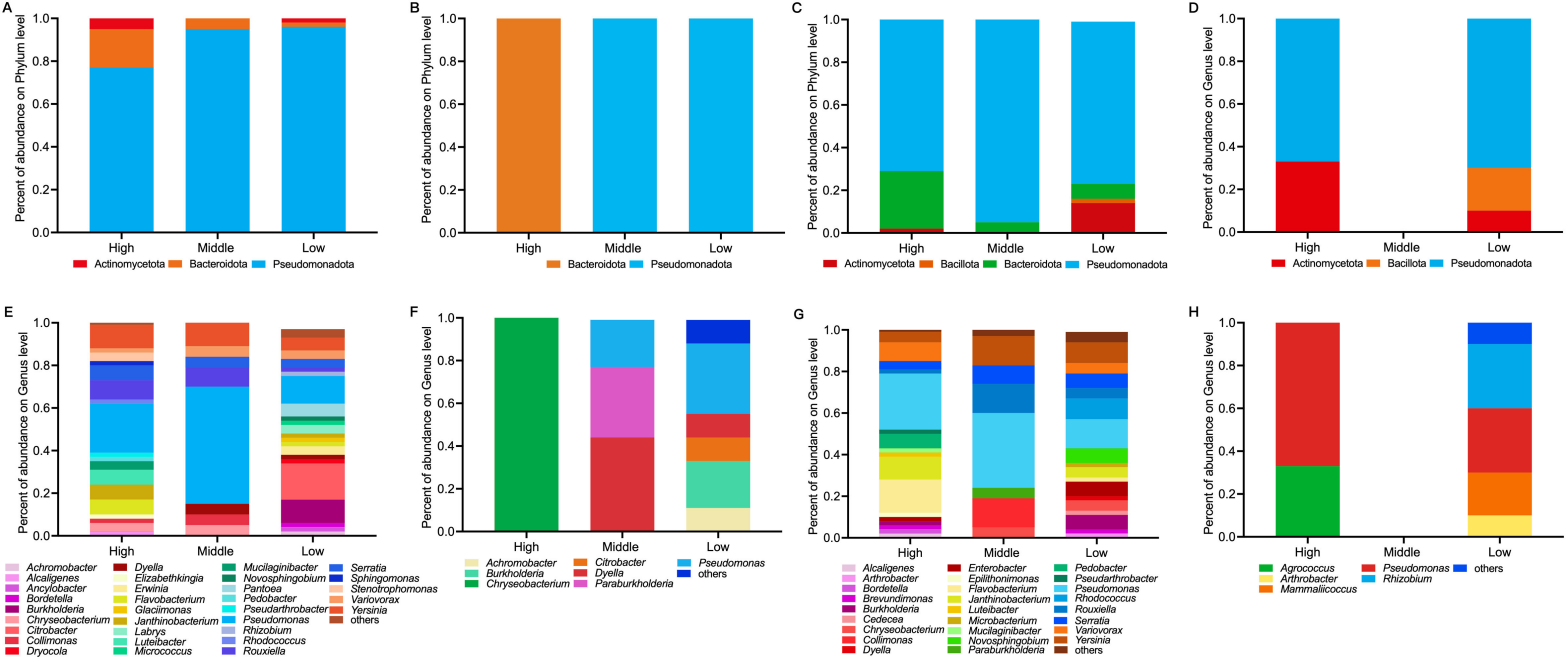
Composition of culturable tuber-associated bacteria. Relative abundance of **(A–D)** phyla and **(E–H)** genera. Panels: **(A, E)** *G. elata* f. *glauca* (tuberosphere soil; n = 8 samples each); **(B, F)** *G. elata* f. *glauca* (endosphere; n = 8 samples each); **(C, G)** *G. elata* f. *elata* (tuberosphere soil; n = 9 samples each); **(D, H)** *G. elata* f. *elata* (endosphere; n = 9 samples each).

Taxonomic composition varied by form and altitude. At the phylum level, *Pseudomonadota* and *Bacteroidota* dominated both forms (**Figures 10A–D**). *G. elata* f. *elata* assemblages included four phyla (*Pseudomonadota*, *Bacteroidota*, *Actinomycetota*, *Bacillota*), while *G. elata* f. *glauca* had three. Altitude effects differed: *G. elata* f. *glauca* phylum richness was highest at high altitude; *G. elata* f. *elata* at low altitude.

At the genus level, 34 and 30 genera were identified for *G. elata* f. *glauca* and *G. elata* f. *elata*, respectively (**Figures 10E–H**). *Pseudomonas* was common and dominant in tuberosphere samples across altitudes. Endophyte composition varied: *G. elata* f. *glauca* endosphere featured *Chryseobacterium* (high), *Dyella* (middle), *Pseudomonas* (low); *G. elata* f. *elata* endophytes included *Pseudomonas*/*Agrococcus* (high), *Rhizobium*/*Mammaliicoccus* (low). Genera shifted across stages (**Supplementary Figure 12**): *G. elata* f. *glauca* tuberosphere richness was highest at high-altitude Medium *Jianma* and low-altitude *Mima*; *G. elata* f. *elata* showed distinct profiles at high-altitude Medium *Jianma* and *Mima*.

#### 3.4.3 Correlation between culturable bacteria, growth traits and bioactive compounds

**Figure 11:**
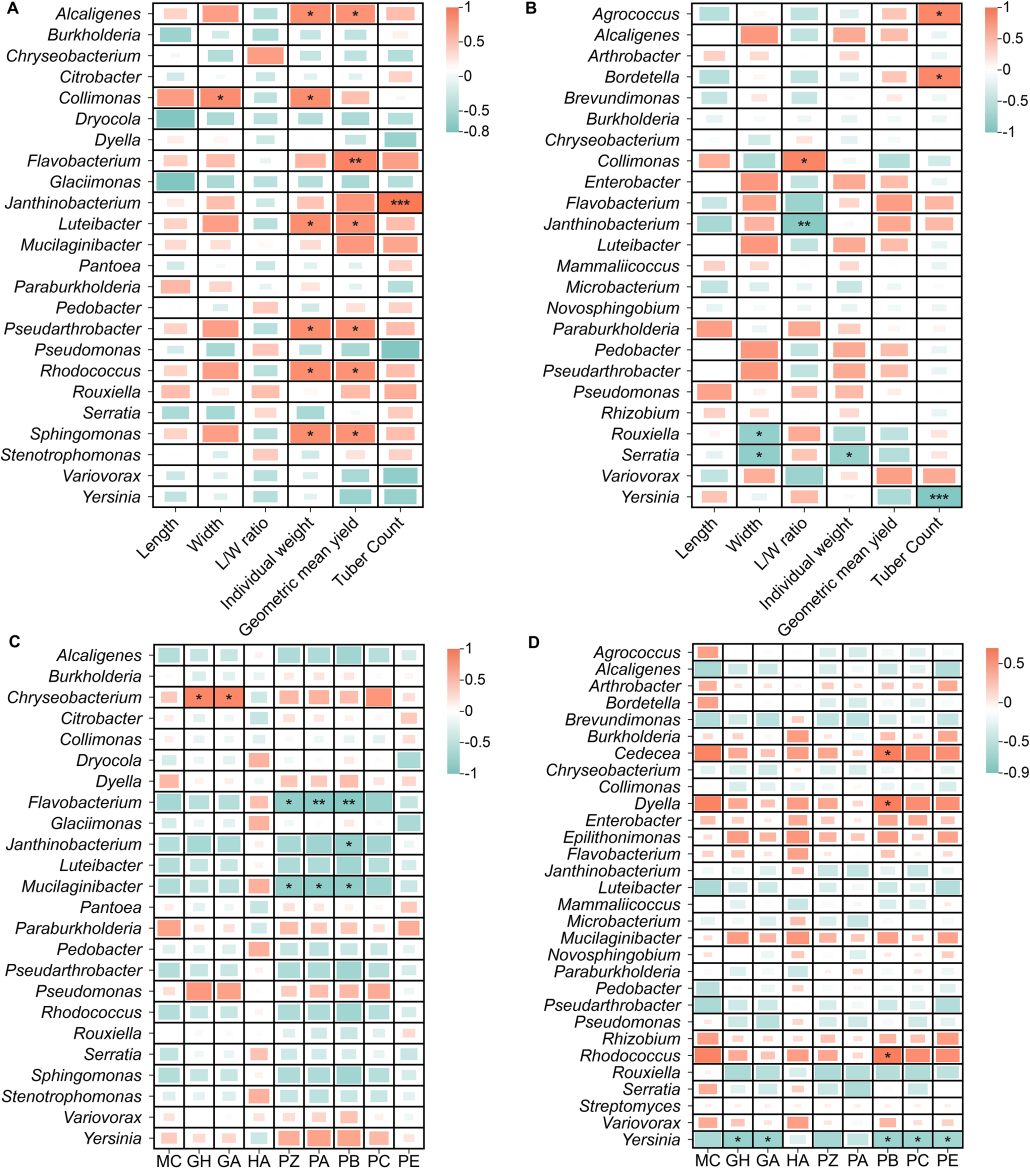
Pearson correlations between culturable bacteria, growth traits **(A, B)** and bioactive components **(C, D)**. **(A, C)** *G. elata* f. *glauca* (n = 8 samples each). **(B, D)** *G. elata* f. *elata* (n = 9 samples each). Abbreviations are defined in **Supplementary Table 1**. ^*^p < 0.05, ^**^p < 0.01, ^***^p < 0.001.

Spearman correlation analysis identified form-specific bacterial genera linked to growth and bioactive traits (*p* < 0.05). For *G. elata* f. *glauca*, seven genera (*Alcaligenes*, *Collimonas*, *Flavobacterium*, *Luteibacter*, *Pseudarthrobacter*, *Rhodococcus*, *Sphingomonas*) correlated positively with yield traits (**Figure 11A**). Three genera showed negative correlations with major bioactive compounds (**Figure 11C**), while *Chryseobacterium* correlated positively with GA. These patterns align with altitudinal trends: yield-associated bacteria link to high-altitude/high-yield, *Chryseobacterium* to middle-altitude/high-quality. For *G. elata* f. *elata*, fewer significant correlations were detected: *Collimonas* (tuber shape), *Agrococcus* and *Bordetella* (tuber number; **Figure 11B**); *Burkholderia* (positive with PB; **Figure 11D**). Yield-associated bacteria linked to high altitude, but no genus strongly correlated with middle-altitude bioactive accumulation.

#### 3.4.4 Associations between yield- and quality-associated culturable bacteria and abiotic/enzyme factors

**Figure 12:**
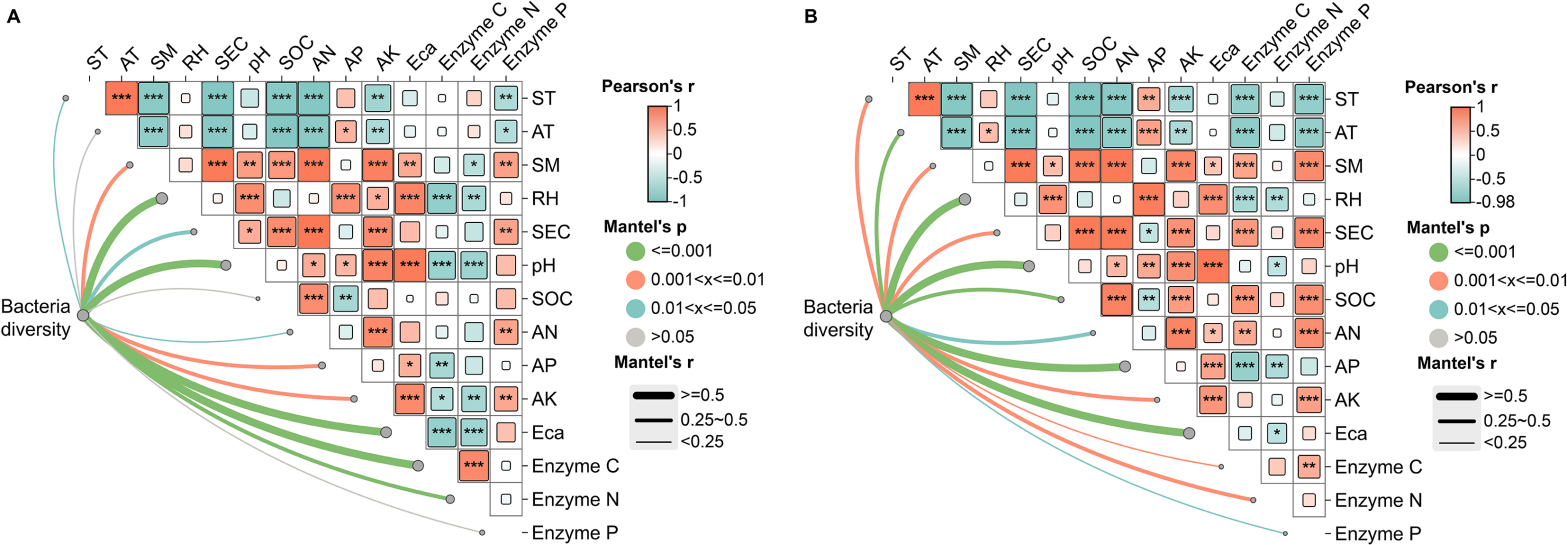
Mantel tests among microclimate, soil properties, soil enzyme activities, and culturable bacteria positively correlated with yield- or quality- associated traits. **(A)** *G. elata* f. *glauca* (n = 8 samples). **(B)** *G. elata* f. *elata* (n = 9 samples). Abbreviations are defined in **Supplementary Table 1**. ^*^p < 0.05, ^**^p < 0.01, ^***^p < 0.001.

Mantel tests were performed to examine associations between yield- and quality-associated culturable bacteria and three categories of factors: microclimate, soil properties, and soil enzymes. For *G. elata* f. *glauca*, yield- and quality-associated bacteria correlated strongly with RH, pH, ECa, and Enzyme C—weakly with AT, SOC, and Enzyme P (**Figure 12A**). For *G. elata* f. *elata*, these bacteria correlated positively with most factors, with weaker links to Enzyme C and P (**Figure 12B**).

**Figure 13:**
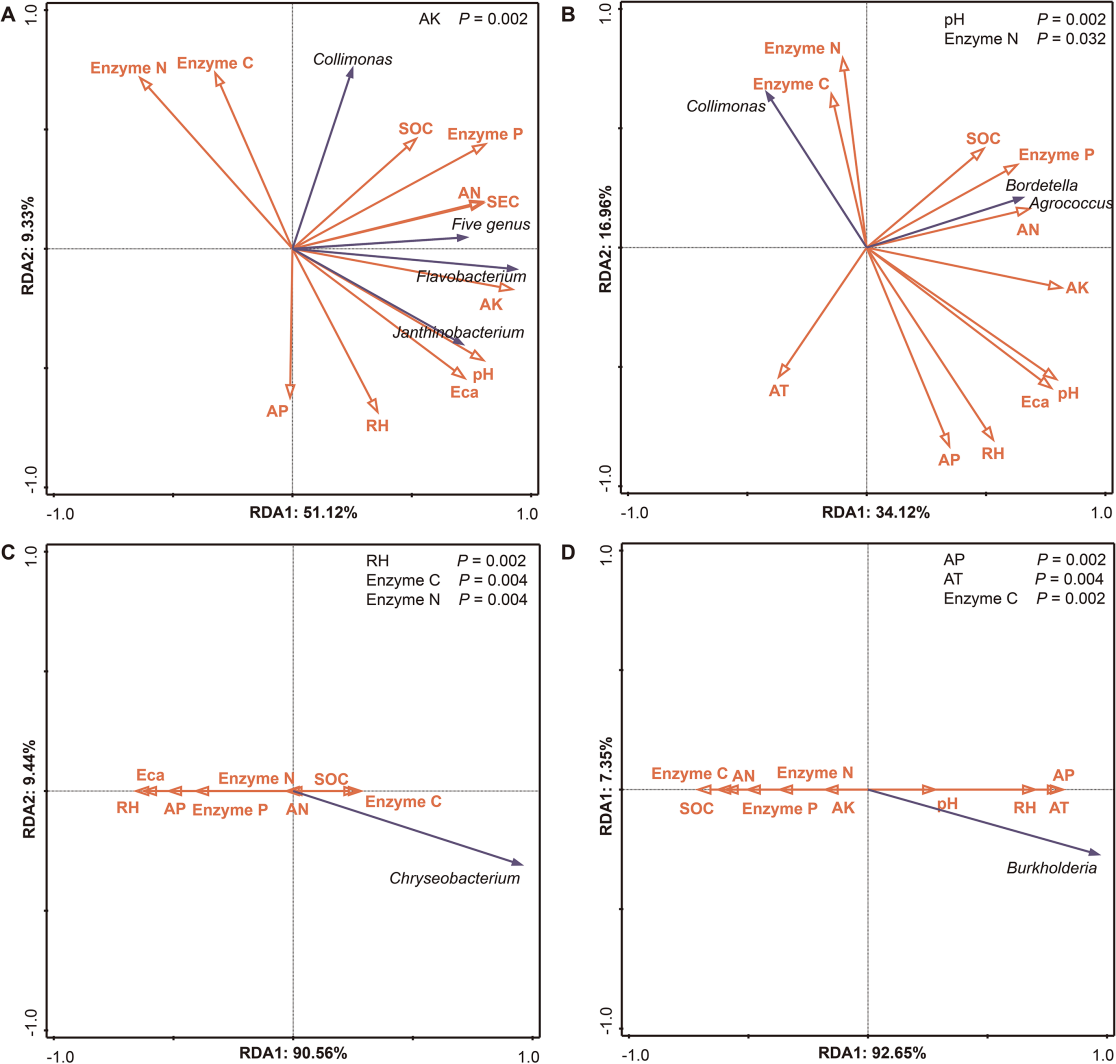
RDA of microclimate, soil properties, soil enzyme activities, and culturable bacteria positively correlated with *G. elata* growth traits and bioactive compounds. **(A, B)** RDA for yield-associated bacterial genera in *G. elata* f. *glauca* (**(A)**; n = 8 samples) and *G. elata* f. *elata* (**(B)**; n = 8 samples). **(C, D)** RDA for quality-associated bacterial genera in *G. elata* f. *glauca* (**(C)**; n = 9 samples) and *G. elata* f. *elata* (**(D)**; n =9 samples). In **(A)**, the five genera *Alcaligenes*, *Luteibacter*, *Pseudarthrobacter*, *Rhodococcus*, and *Sphingomonas* are represented collectively by a single arrow. Abbreviations are defined in **Supplementary Table 1**. *p* < 0.05.

RDA further clarified these patterns: *G. elata* f. *glauca* yield-associated bacteria were primarily associated with AK, which explained 43.7% of variation (*p* = 0.002; **Figure 13A**); the quality-associated genus *Chryseobacterium* in *G. elata* f. *glauca* showed significant correlations with RH (39%) and Enzyme C (35.1%) (*p* < 0.05), with an inverse correlation to Enzyme N (**Figure 13C**); *G. elata* f. *elata* yield-associated bacteria were linked to pH (26.6%) and Enzyme N (12.1%) as key correlative factors (**Figure 13B**); the quality-associated genus *Burkholderia* in *G. elata* f. *elata* was primarily associated with AP, which explained 61.6% of variation (*p* = 0.002), with an inverse correlation to Enzyme C (**Figure 13D**).

### 3.5 Associations of growth with the abiotic environment, soil enzymes, and culturable bacteria in two *G. elata* forms

**Figure 14:**
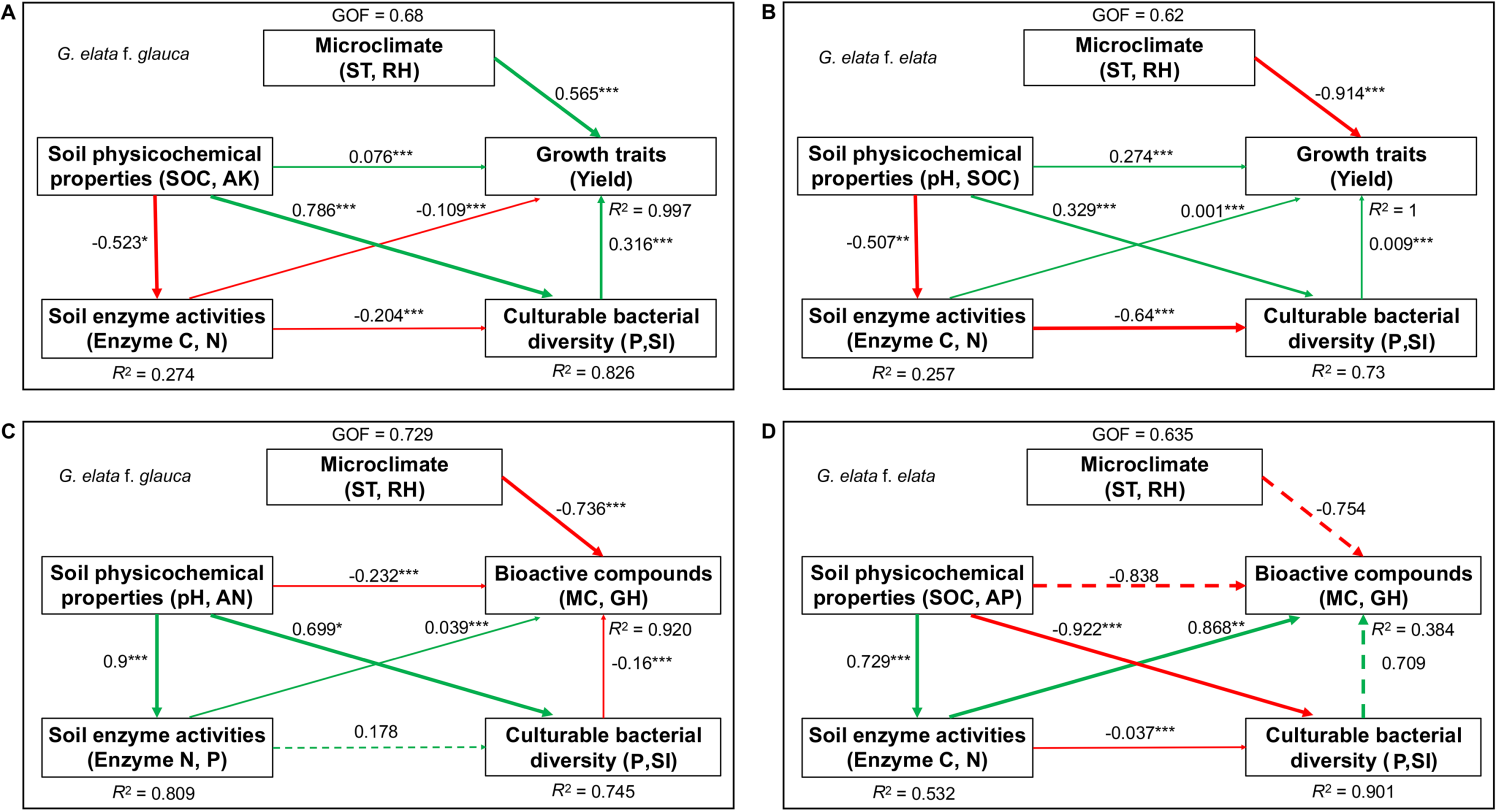
PLS-SEM models depicting factors associated with growth traits and bioactive compounds accumulation in *G. elata*. **(A)** *G. elata* f. *glauca* growth (n = 8 samples); **(B)** *G. elata* f. *elata* growth (n = 9 samples); **(C)** *G. elata* f. *glauca* bioactive compounds accumulation; **(D)** *G. elata* f. *elata* bioactive compounds accumulation. Solid green arrows indicate significant positive paths, and solid red arrows indicate significant negative paths, with arrow thickness corresponding to path coefficient magnitude. Dashed arrows represent non-significant paths. *D*, Simpson index; P, Relative abundance of culturable bacterial genera associated with yield or quality; MC, total concentration of all six compounds; GH, Sum of GA and HBA. Other abbreviations are defined in **Supplementary Table 1**. ^*^p < 0.05, ^**^p < 0.01, ^***^p  < 0.001.

PLS-SEM explored multivariate correlative relationships (all models: VIF < 3, GoF: 0.62-0.729). For *G. elata* f. *glauca*, yield (*R*² = 0.997, GoF = 0.68; **Figure 14A**) showed a direct positive association with microclimate (ST, RH) (path coefficient = 0.565, *p* < 0.001), while soil properties (SOC, AK) were positively linked to culturable bacterial diversity (0.786, *p* < 0.001)—a factor that strongly correlated with yield (0.315, *p* < 0.001); soil properties were negatively linked to enzymes (-0.523, *p* < 0.001), which showed a weak negative correlation with yield (-0.109, *p* < 0.001). For *G. elata* f. *glauca* bioactive compounds (*R*² = 0.92, GoF = 0.729; **Figure 14C**), only microclimate showed a direct negative association (-0.736, *p* < 0.01); soil properties (pH, AN) were positively linked to enzymes (0.9, *p* < 0.001) and bacterial diversity (0.699, *p* < 0.001), but these factors had no significant direct links to bioactive compounds.

For *G. elata* f. *elata*, yield (*R*² = 1, GoF = 0.62; **Figure 14B**) exhibited a dominant negative association with microclimate (-0.914, *p* < 0.001), while soil properties (pH, SOC) showed a weak positive correlation with yield (0.274, *p* < 0.001), were negatively linked to enzymes (-0.507, *p* < 0.01), and positively associated with bacterial diversity (0.329, *p* < 0.05); enzymes were strongly negatively linked to bacterial diversity (-0.640, *p* < 0.001), but neither factor directly correlated with yield. For *G. elata* f. *elata* bioactive compounds (*R*² = 0.384, GoF = 0.635; **Figure 14D**), soil properties (AP, SOC) were positively linked to enzymes (0.729, *p* < 0.001)—a factor that strongly correlated with bioactive compounds (0.867, *p* < 0.01)—while soil properties were negatively correlated with bioactive compounds (-0.838, *p* < 0.05); microclimate and bacterial diversity showed large path coefficients (path coefficient > 0.7) but no significant direct associations. These results highlight form-specific correlative pathways. Soil properties act as a key indirect factor across both forms, shaping plant performance via enzymes and culturable bacterial diversity.

## 4 Discussion

### 4.1 Study design and integrated multi-factor analysis

Previous studies focused on single-form yield/quality differences across habitats or multi-form comparisons in the same habitat (Luo et al., 2022; Tian et al., 2020; Yang et al., 2024; Shi et al., 2025), simplistically treating habitat as a single variable. High-quality Chinese medicinal herbs (genuine medicinal materials) form via interactions of genotype, environment, and cultivation (Zhang C. C. et al., 2023; Zhang W. et al., 2023). *G. elata* is environmentally sensitive, with five forms classified by morphology (Editorial Committee of the Flora of China, Chinese Academy of Sciences, 1999); three common forms (*G. elata* f. *glauca*, f. *viridis*, f. *elata*) exhibit vertical distribution (f. *glauca* > f. *viridis* > f. *elata*). This study compared forms at the same altitude to maximize variable control, using mixed linear models and PLS-SEM to analyze microclimate, soil properties, enzymes, and culturable bacteria—providing a comprehensive assessment of growth- and quality-associated factors.

Previous *G. elata* quality standards focused on *Jianma* stage bioactive content (Li et al., 2024; Yang et al., 2023), but compound accumulation shows stage specificity (Zhu et al., 2024)—confirmed here: “GA and total bioactive compounds were higher in *G. elata* f. *glauca* Large *Baima* and *G. elata* f. *elata Mima* than other stages.” We subdivided tubers into seven stages (three *Jianma* stages, three *Baima* stages, and the *Mima* stage) and measured growth traits and bioactive content for each. Mixed linear models, RDA, and heatmaps showed altitude, form, and stage correlate significantly with growth traits—stage having the strongest explanatory power (66.7%). To our knowledge, this is the first study analyzing synergistic effects of altitude, form, and stage on *G. elata* growth and quality, providing a reference for optimal harvest timing.

### 4.2 Diversity and correlative characteristics of culturable tuber-associated microbes

*G. elata* is a mycoheterotrophic plant dependent on germination fungi (*Mycena* spp.) and *Armillaria* for nutrition (Xu, 1993; Guo et al., 1999). For practical applications, we used culture-dependent methods to isolate culturable microbes—no symbiotic *Mycena* or *Armillaria* were identified, likely due to isolation constraints: germination fungi are typically isolated from protocorms (Xu, 1993; Xu and Guo, 1989; Guo et al., 1999), while *Armillaria* requires tissue near tuber surfaces or rhizomorphs (Xu, 1993; Cheng et al., 2023; Chen et al., 2023). Our focus on *Jianma*/*Baima*/*Mima* stages missed early germination stages, and the homogenization method may have damaged *Armillaria*—highlighting the need for targeted isolation strategies for these symbionts.

The limited fungal isolates (mostly pathogenic and saprophytic) likely reflect *Armillaria*’s dominance in the cultivation system, suppressing other fungi (Xu et al., 2020; Zhang et al., 2022; Wang YH, et al., 2023). We obtained 466 bacterial isolates (164 OTUs), indicating high culturable bacterial richness—consistent with reports that *G. elata* cultivation increases bacterial diversity (Ye et al., 2025). Correlation analysis identified form-specific bacteria linked to yield/quality, with abiotic/enzyme effects varying by form (*p* < 0.05). For instance, AK was strongly associated with yield-linked bacteria in *G. elata* f. *glauca*, accounting for 43.7% of the observed variation. Meanwhile, RH (39%) and Enzyme C (35.1%) correlated with the quality-associated genus *Chryseobacterium* in this form. In contrast, yield-linked bacteria in *G. elata* f. *elata* showed an association with pH (26.6%), and its quality-associated genus *Burkholderia* was linked to AP (61.6%). These correlation patterns align with the findings of previous studies (Jiang et al., 2025; Ye et al., 2025) that highlight the habitat-dependent link between soil factors and tuber microbial assemblages in medicinal orchids. However, these yield/quality-associated bacteria require further functional validation via inoculation experiments to confirm beneficial effects.

### 4.3 Regulatory mechanisms of tuber yield and bioactive compound accumulation

Mixed linear models identified altitude as the dominant factor associated with yield: yields at the high-altitude site (1953 m) were significantly higher than those at the middle and low altitudes—consistent with Luo et al. (2023), who identified 1800 m as optimal for wild-simulated cultivation of *G. elata* f. *glauca*. PLS-SEM revealed form-specific yield pathways: microclimate was the core direct factor, with opposite effects—positive for *G. elata* f. *glauca* (adapted to high-altitude low temperature and large diurnal temperature differences) and negative for *G. elata* f. *elata* (preferring warm, humid middle-low altitudes). *G. elata* f. *glauca* had a unique indirect pathway: soil properties promoted yield via culturable bacterial diversity, highlighting soil-microbe interactions in high-yield formation.

Altitude exerts divergent effects on the accumulation of bioactive compounds in medicinal plants. For some species, altitude promotes compound synthesis—such as triterpenes in *Codonopsis pilosula* var. *modesta* (Wang et al., 2025) and dendrobine in *Dendrobium nobile* (Li L, et al., 2022). For others, altitude inhibits compound accumulation, including glycyrrhizin in *Glycyrrhiza glabra* (Eghlima et al., 2025) and anthraquinones in wild *Rheum tanguticum* (Li et al., 2025). Heatmap analysis revealed that both *G. elata* forms had enriched bioactive compounds at the middle altitude (1653 m), with distinct stage-specificity. Specifically, the peak accumulation occurred at the Large *Baima* stage in *G. elata* f. *glauca* and at the *Mima* stage in *G. elata* f. *elata*. At these optimal stages, the GA content and total bioactive compound content in *G. elata* f. *glauca* were 3.33-fold and 1.72-fold higher than those in *G. elata* f. *elata*, respectively (**Supplementary Table 14-15**). Notably, asexual propagation limited the availability of *G. elata* f. *glauca Mima* samples, preventing a complete comparison of bioactive compound accumulation at this juvenile stage between the two forms. Based on previous studies reporting *Mima*-stage bioactive enrichment in *G. elata* (Zhu et al., 2024), we hypothesize that *G. elata* f. *glauca Mima* may also exhibit high bioactive compound concentrations—this requires validation in future studies via sexual cultivation to ensure sufficient *Mima*-stage samples for *G. elata* f. *glauca*.

PLS-SEM revealed divergent quality pathways: *G. elata* f. *glauca* bioactive compounds were directly negatively affected by microclimate; *G. elata* f. *elata* relied on the indirect “soil properties → enzymes” correlative pathway. Notably, microclimate and soil factors had opposite effects on yield and quality, suggesting a growth-quality resource allocation trade-off (Xie et al., 2019)—providing a theoretical basis for “synergistic regulation of high yield and quality”. From a physiological perspective, GA and parishins interconvert via enzymatic reactions (Yan et al., 2025; Li JY, et al., 2022) and non-enzymatic degradation (Tian et al., 2017), For example, *GeCXE9* hydrolyzes PA to GA. *G. elata* lacks key nitrogen metabolism genes, relying on symbiotic fungi for nitrogen (Yuan et al., 2025), suggesting GA synthesis may depend on fungal-provided nitrogen precursors. Combined with our results, enzymes and bacterial diversity may regulate quality by influencing symbiotic metabolism and bioactive transformation—providing a direction for future molecular mechanism studies.

### 4.4 Limitations and future perspectives

This study constructed a multi-dimensional model integrating abiotic factors, enzyme activities, microbial diversity, growth traits, and bioactive compounds, but it has several limitations. First, data integrity was constrained by the delayed start of meteorological monitoring (two months post-planting), which missed the early-stage *Armillaria* colonization conditions; additionally, asexual propagation limited the availability of *G. elata* f. *glauca Mima* samples, resulting in bioactive compound analysis covering 6 stages for this form versus 7 stages for *G. elata* f. *elata*. Second, the experimental design was restricted to a single growing season in the Qinba Mountains, meaning the conclusions about altitude effects need to be verified by multi-year and cross-regional experiments (e.g., in the Yunnan-Guizhou Plateau) to confirm their stability and generalizability. Third, the culture-dependent microbial analysis method overlooked unculturable microbes, potentially excluding key symbiotic taxa that may contribute to tuber growth and quality.

Future research should focus on three key directions to address these limitations. First, cultivation and monitoring protocols need optimization: sexual cultivation should be adopted to ensure sufficient *G. elata* f. *glauca Mima* samples, and environmental monitoring equipment should be deployed pre-planting to achieve full-cycle tracking of microclimate and soil conditions. Second, the experimental scale should be expanded by increasing sample sizes, conducting multi-year and multi-site experiments, and comparing the Qinba Mountains with other major *G. elata*-growing regions like the Yunnan-Guizhou Plateau to enhance the generalizability of the findings. Third, innovative analytical techniques should be integrated: metagenomics can be used to characterize the full microbial community, greenhouse inoculation experiments can validate the functional roles of key bacteria, and combined metabolomics and transcriptomics can clarify the enzyme-regulated transformation pathways of bioactive compounds. Additionally, future studies should explore adaptive planting strategies in the context of climate change, targeting key environmental factors such as May–July solar radiation, October–November precipitation, and the coldest month minimum temperature (Hao et al., 2024; Xi et al., 2025). Ultimately, interdisciplinary integration will construct a precise “environment-microbe-metabolism” regulatory system for *G. elata*, supporting quality improvement, efficiency enhancement, and sustainable industry development.

## 5 Conclusion

Altitude correlates with yield and bioactive compound accumulation in two *G. elata* forms, potentially via associations with microclimate, soil properties, enzyme activities, and culturable bacterial assemblages. High altitude (1953 m) associates with higher yield, while middle altitude (1653 m) links to bioactive enrichment—with *G. elata* f. *glauca* optimal at Large *Baima* and *G. elata* f. *elata* at *Mima*. Soil pH and carbon-acquiring enzyme activity are form-specific key correlative factors for quality, and culturable bacterial diversity correlates with promoted growth but inhibited bioactive accumulation. These findings provide targeted guidance for selecting suitable altitudes and harvest stages to balance high yield and quality in *G. elata* cultivation.

## Data Availability

The datasets presented in this study can be found in online repositories. The names of the repository/repositories and accession number(s) can be found below: https://www.ncbi.nlm.nih.gov/genbank/, PX515666–PX515829.
