# Supplementary material for: Altitude modulates growth and bioactive compounds in two Gastrodia elata forms through the microenvironment and soil microbes
Source: Front Plant Sci. 2026 Mar 5;17:1734174. doi: 10.3389/fpls.2026.1734174 (PMC12999896; doi:10.3389/fpls.2026.1734174)
Supplement: Supplementary file 1 [file DataSheet1.docx]

Supplementary Material

# Supplementary Figures and Tables

## Supplementary Figures


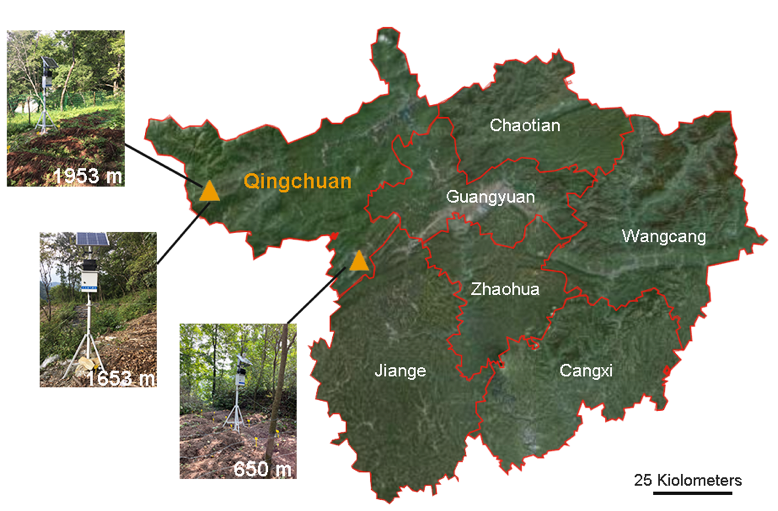


**Supplementary Figure 1** Geographical location of the three study sites in Guangyuan City, Sichuan Province, China. *Cultivation sites and administrative division names were independently annotated by the authors. The territorial depiction is complete and accurate.*


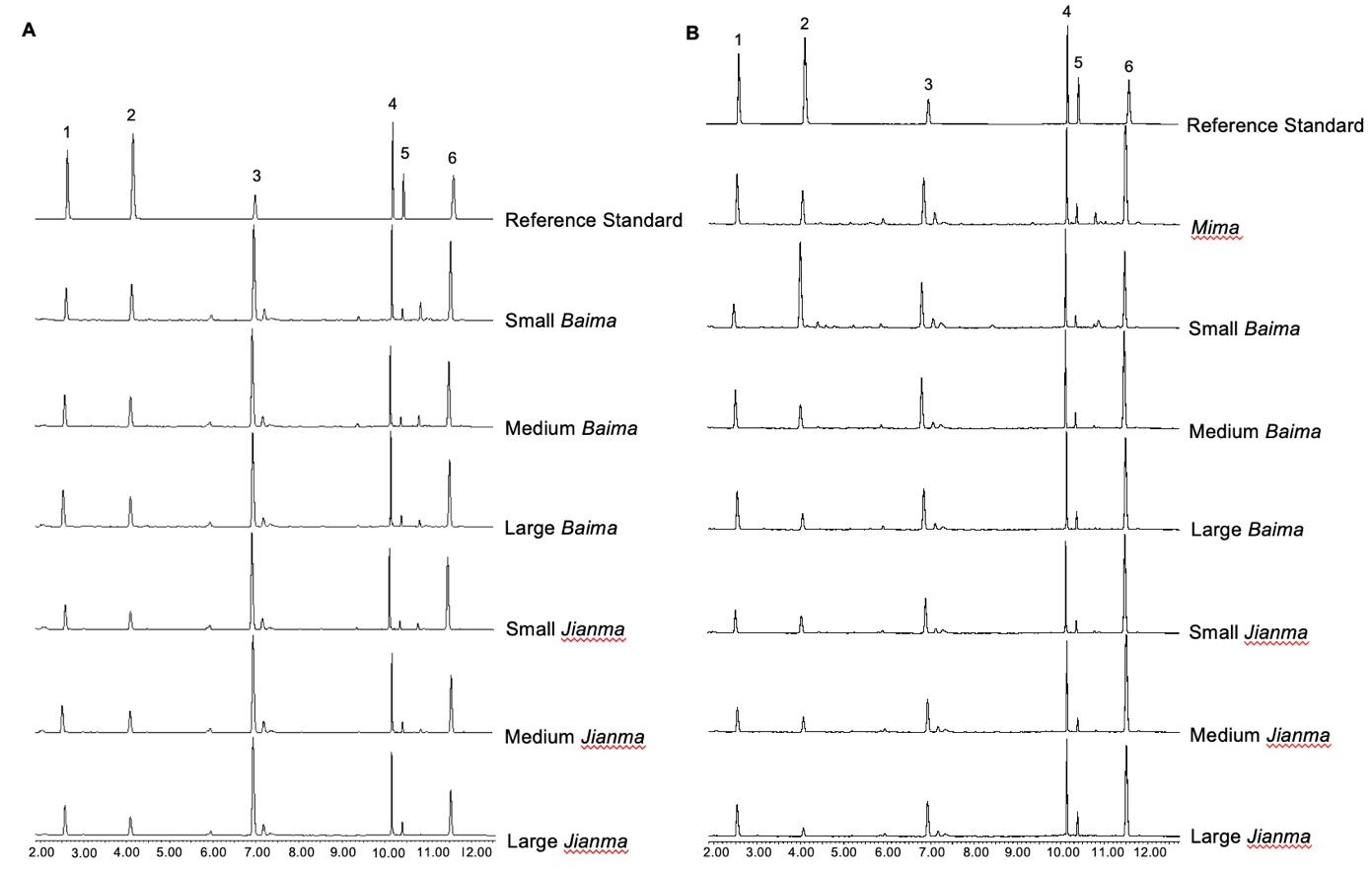


**Supplementary Figure 2.** UPLC chromatograms of six bioactive compounds in tubers of two *G. elata* forms cultivated at a high-altitude site (1953 m). **(A)** *G. elata* f. *glauca* across six developmental stages; **(B)** *G. elata* f. *elata* across seven developmental stages. Identified peaks: 1, gastrodin; 2, *p*-hydroxybenzyl alcohol; 3, parishin E; 4, parishin B; 5, parishin C; 6, parishin A.


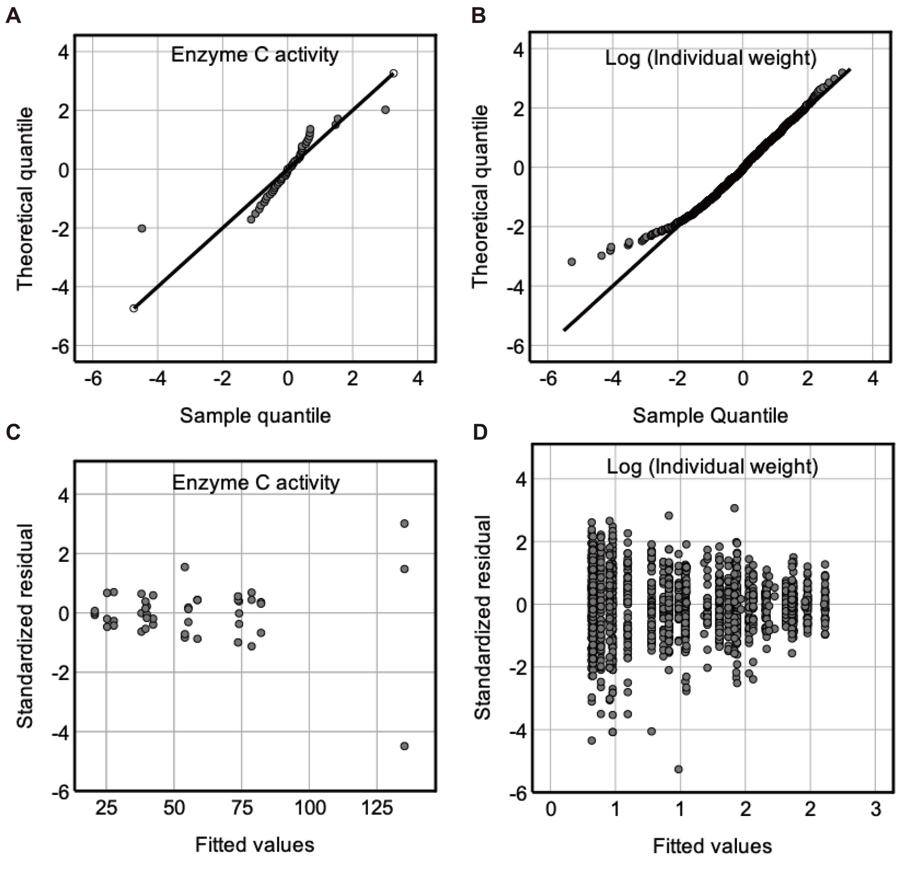


**Supplementary Figure 3.** Diagnostic plots for two representative LMMs (n = 3 cultivation beds). **(A, B)** Quantile-quantile (Q-Q) plots of standardized residuals versus the theoretical normal quantiles. The close alignment of points to the dashed reference line indicates no substantial deviation from normality. **(C, D)** Plots of standardized residuals versus model-fitted values. The random scatter of points without discernible patterns (e.g., funnel shape) supports the assumption of homoscedasticity (constant variance of residuals). These LMMs predict Enzyme C activity and log (Individual weight) based on altitude, form, developmental stage, and their interactions, with a random intercept for cultivation bed. The diagnostic procedures illustrated here were consistently applied to all LMMs reported in the study.


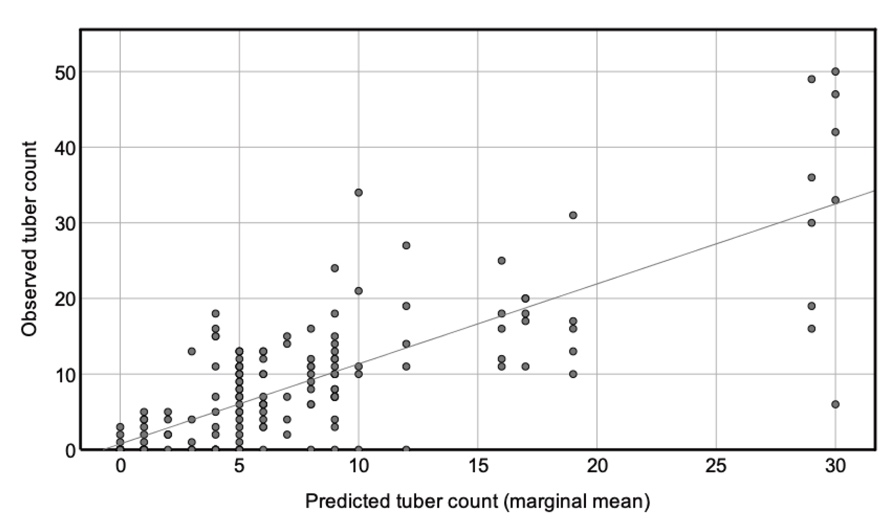


**Supplementary Figure 4.** Diagnostic plot for the GLMM of tuber count (n = 5 cultivation beds). Plot of observed tuber counts versus model-predicted counts. Reasonably symmetric distribution of points around the dashed line of unity (y = x) indicates an acceptable model fit for the count data. The coefficient of determination (*R*²) is 0.619. This GLMM uses a negative binomial distribution with a log link function to predict tuber count per plant.


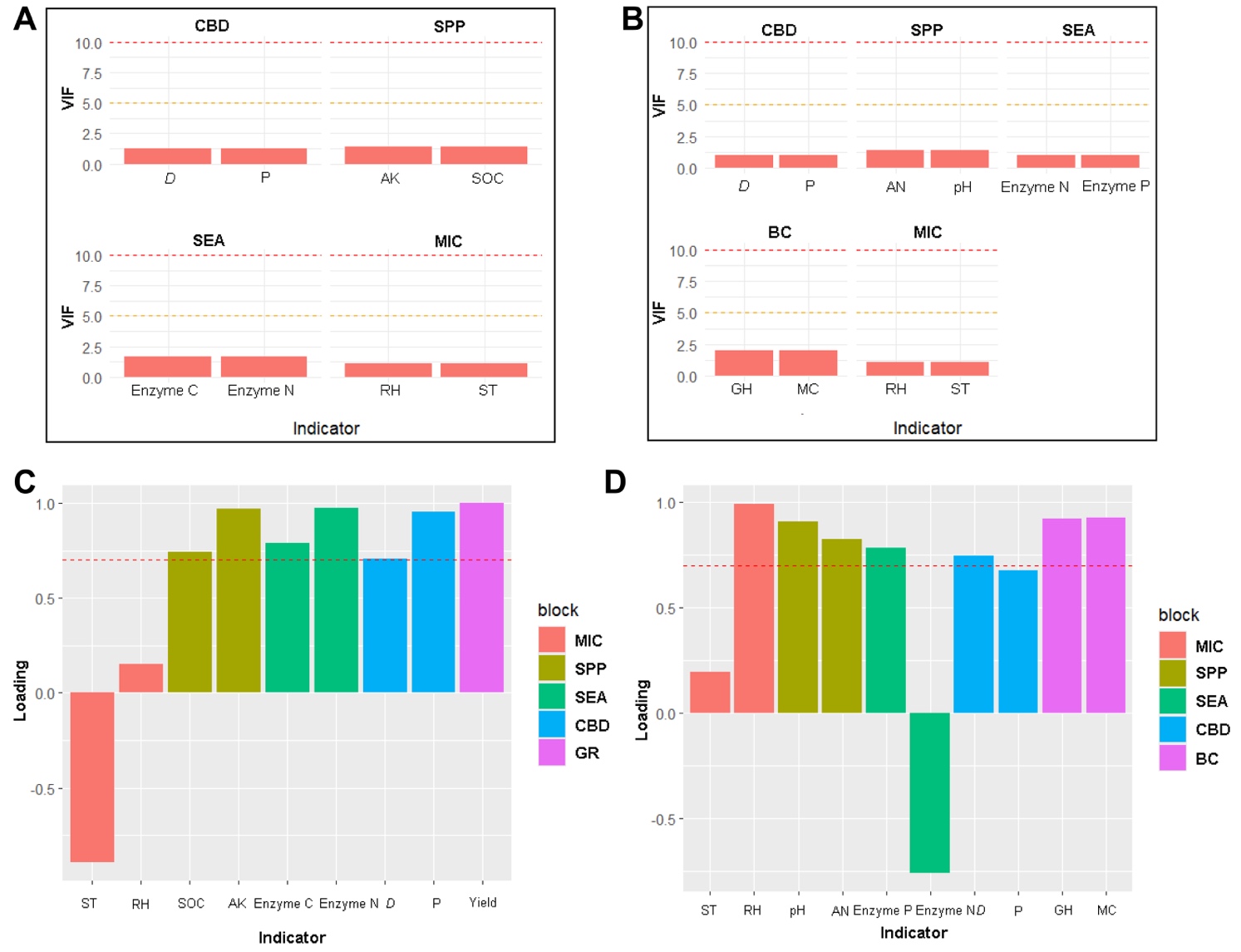


**Supplementary Figure 5.** VIF plots and indicator loading plots for PLS-SEM measurement models of *G. elata* f. *glauca* growth and bioactive compound accumulation. (n = 8 samples). **(A)** VIF plot for the growth-related model; **(B)** VIF plot for the bioactive compound accumulation-related model; **(C)** Indicator loading bar plot for the growth-related model; **(D)** Indicator loading bar plot for the bioactive compound accumulation-related model. In VIF plots, the red line denotes the severe collinearity threshold (VIF = 10), and the yellow line denotes the high collinearity threshold (VIF = 5); all indicators in the models had VIF values < 3, confirming acceptable collinearity. In indicator loading plots, the dashed line marks the |0.7| reliability threshold for manifest variables on their respective latent constructs (microclimate, soil properties, soil enzyme activities, and culturable bacterial diversity). Abbreviations: MIC, Microclimate; SPP, soil physicochemical properties; SEA, Soil enzyme activities; CBD, Culturable bacterial diversity; GR, Growth traits; BC, Bioactive compounds. Other abbreviations are defined in **Supplementary Table 1**.


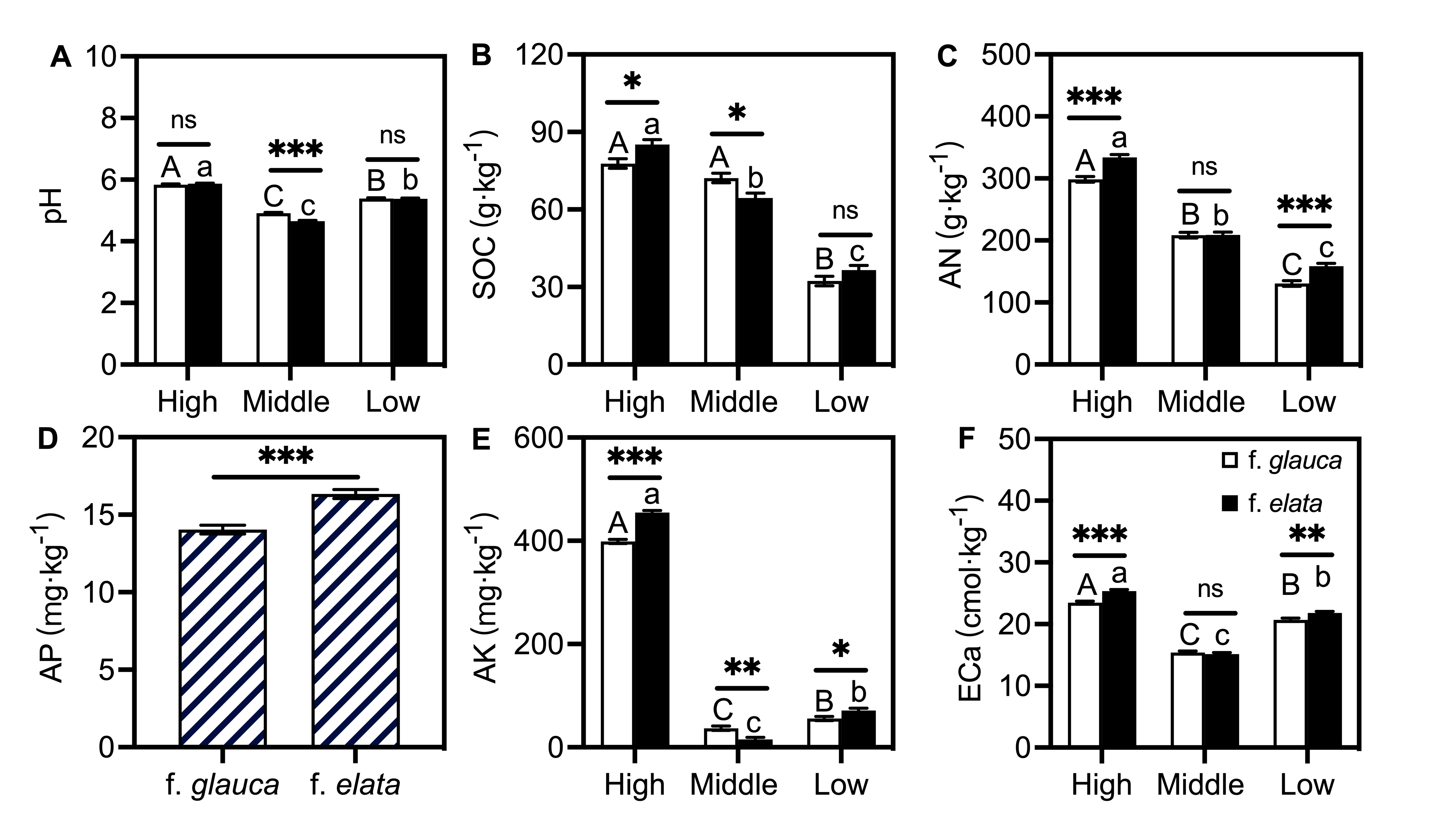


**Supplementary Figure 6.** Variations in bulk soil physicochemical properties across three altitudes for two *G. elata* forms. Data are shown as mean ± SE (n = 3 cultivation beds). Different uppercase (f. *glauca*) and lowercase (f. *elata*) letters denote significant differences among altitudes (*p* < 0.05); asterisks mark significant differences between the two forms at a given altitude (Šidák's test; ^*^*p* < 0.05, ^**^*p* < 0.01, ^***^*p* < 0.001). Abbreviations are defined in **Supplementary Table 1**.


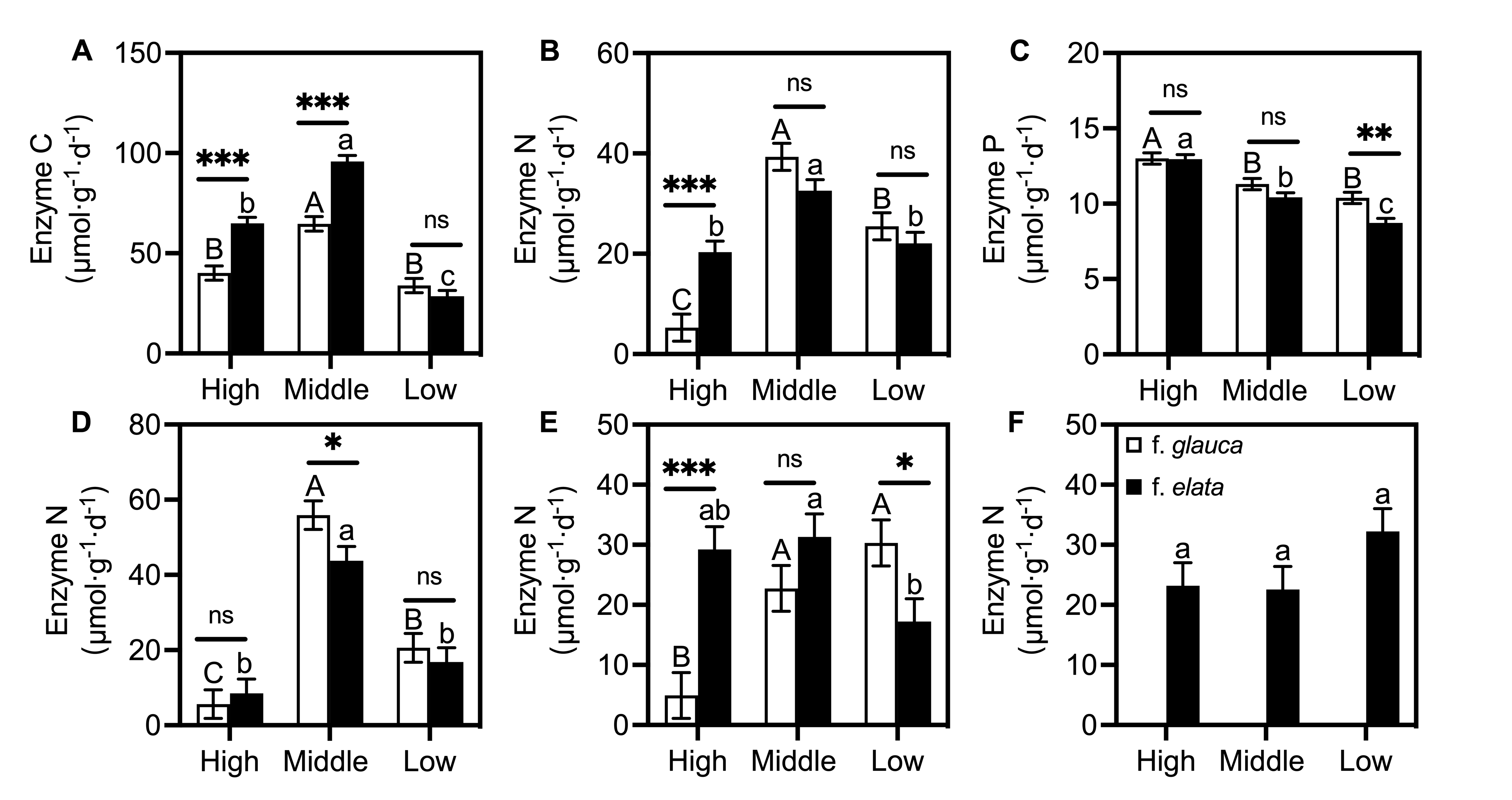


**Supplementary Figure 7.** Relative effects of *G. elata* form, developmental stage, and altitude on tuberosphere enzyme activities. Mean ± SE (n = 3 cultivation beds). **(A-C)** Form × stage interactions for C-, N-, and P-acquiring enzymes. **(D-F)** Altitude × form × stage interactions for N-acquiring enzyme. Different uppercase (f. *glauca*) and lowercase (f. *elata*) letters denote significant differences among altitudes (*p* < 0.05); asterisks highlight significant differences between forms under specific altitude-stage conditions (Šidák's test; ^*^*p* < 0.05, ^**^*p* < 0.01, ^***^*p* < 0.001). Full enzyme names are provided in **Supplementary Table 1**.


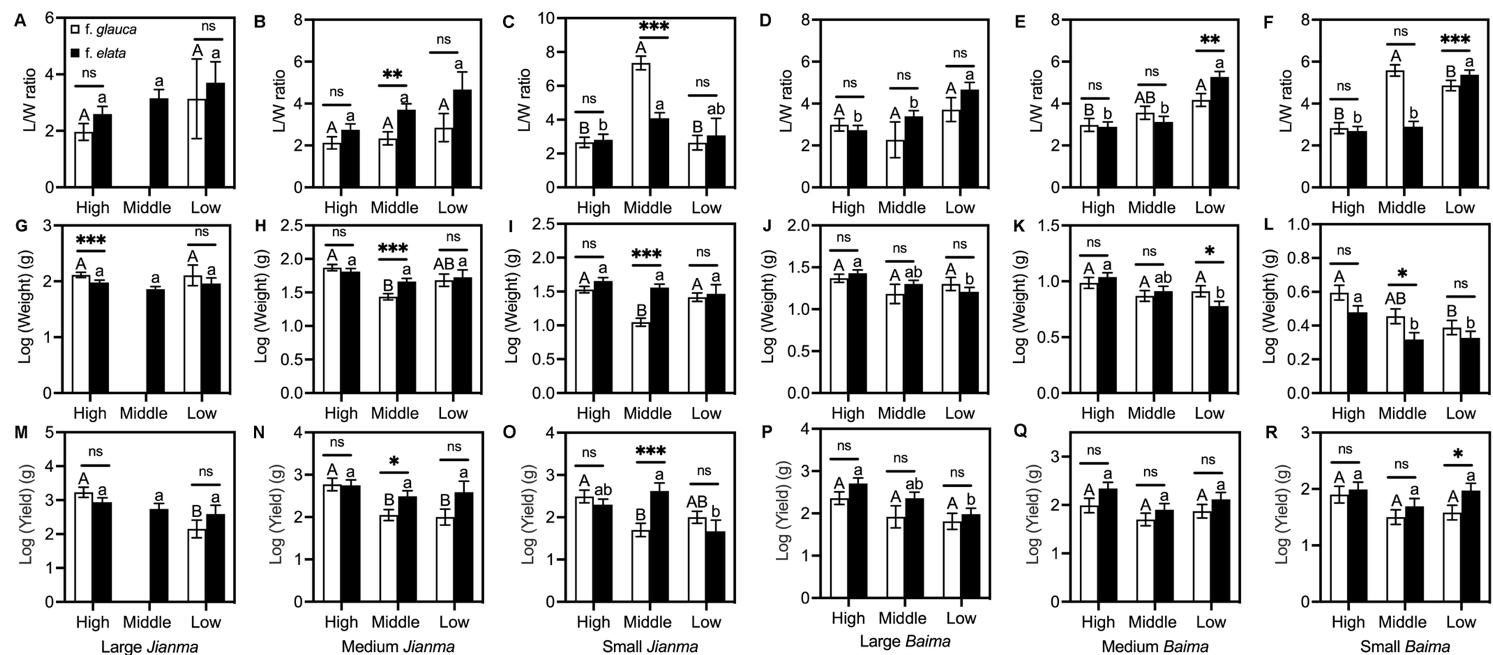


**Supplementary Figure 8.** Developmental changes in tuber length-to-width ratio, log-transformed weight, and log-transformed yield across three altituds in two *G. elata* forms. Values are presented as means ± SE (n = 5). Different uppercase (f. *glauca*) and lowercase (f. *elata*) letters denote significant differences among altitudes (*p* < 0.05); asterisks indicate significant differences between forms at a specific altitude (Šidák's test: ^*^*p* < 0.05, ^**^*p* < 0.01, ^***^*p* < 0.001).


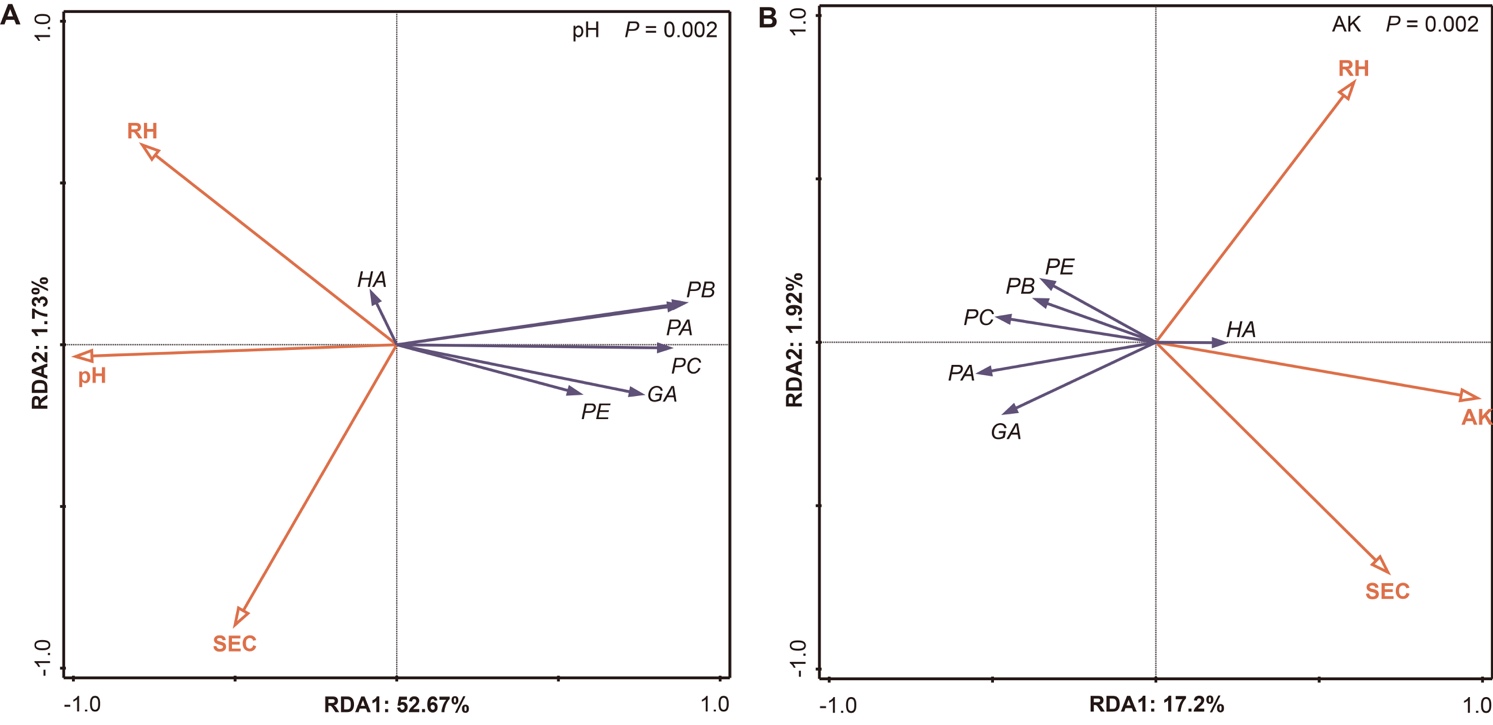


**Supplementary Figure 9.** RDA of bioactive compounds in relation to microclimate and soil properties. **(A)** *G. elata* f. *glauca* (n = 18 samples). **(B)** *G. elata* f. *elata* (n = 21 samples). Abbreviations are defined in **Supplementary Table 1**. *p* < 0.05.


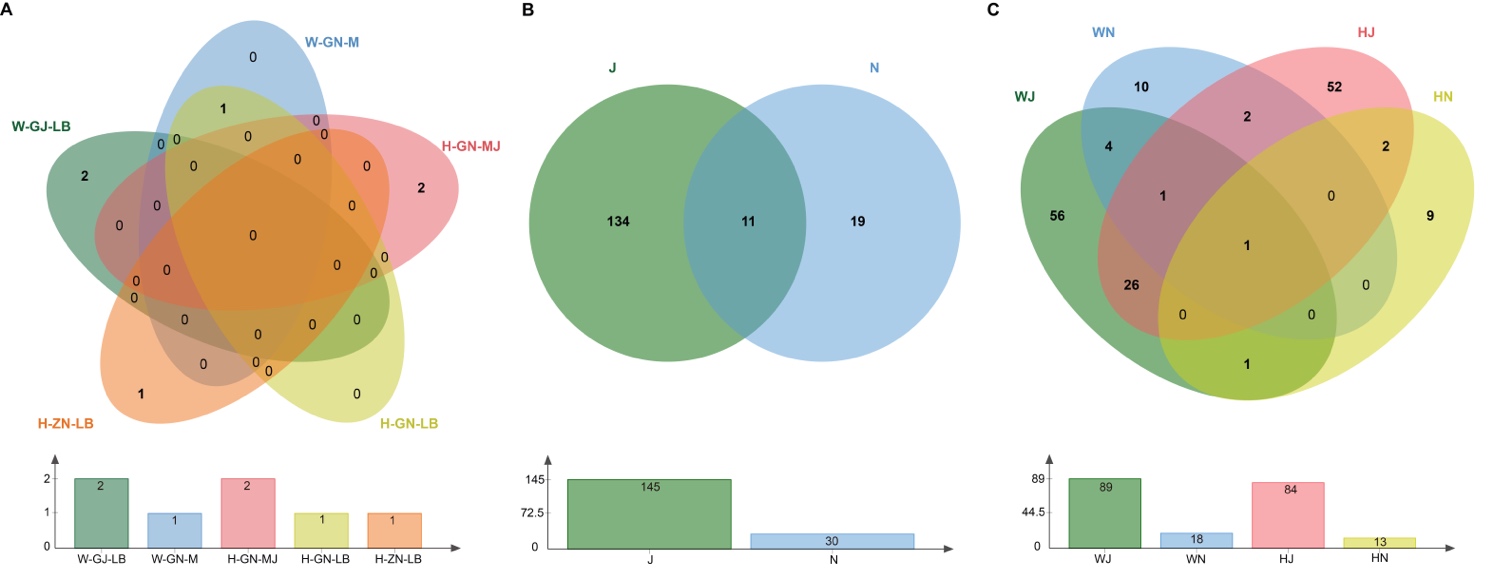


**Supplementary Figure 10.** Venn diagrams of OTU distribution for culturable tuber-associated fungi and bacteria (n = 17 samples each). **(A)** Fungal OTUs. **(B)** Bacterial OTUs partitioned by habitat (J, tuberosphere soil; N, endosphere). **(C)** Bacterial OTUs partitioned by *G. elata* form (W, f. *glauca*; H, f. *elata*). Sample codes for panel A: Form, altitude (G, high; Z, middle), habitat, and developmental stage (MJ, Medium *Jianma*; LB, Large *Baima*; M, *Mima*).


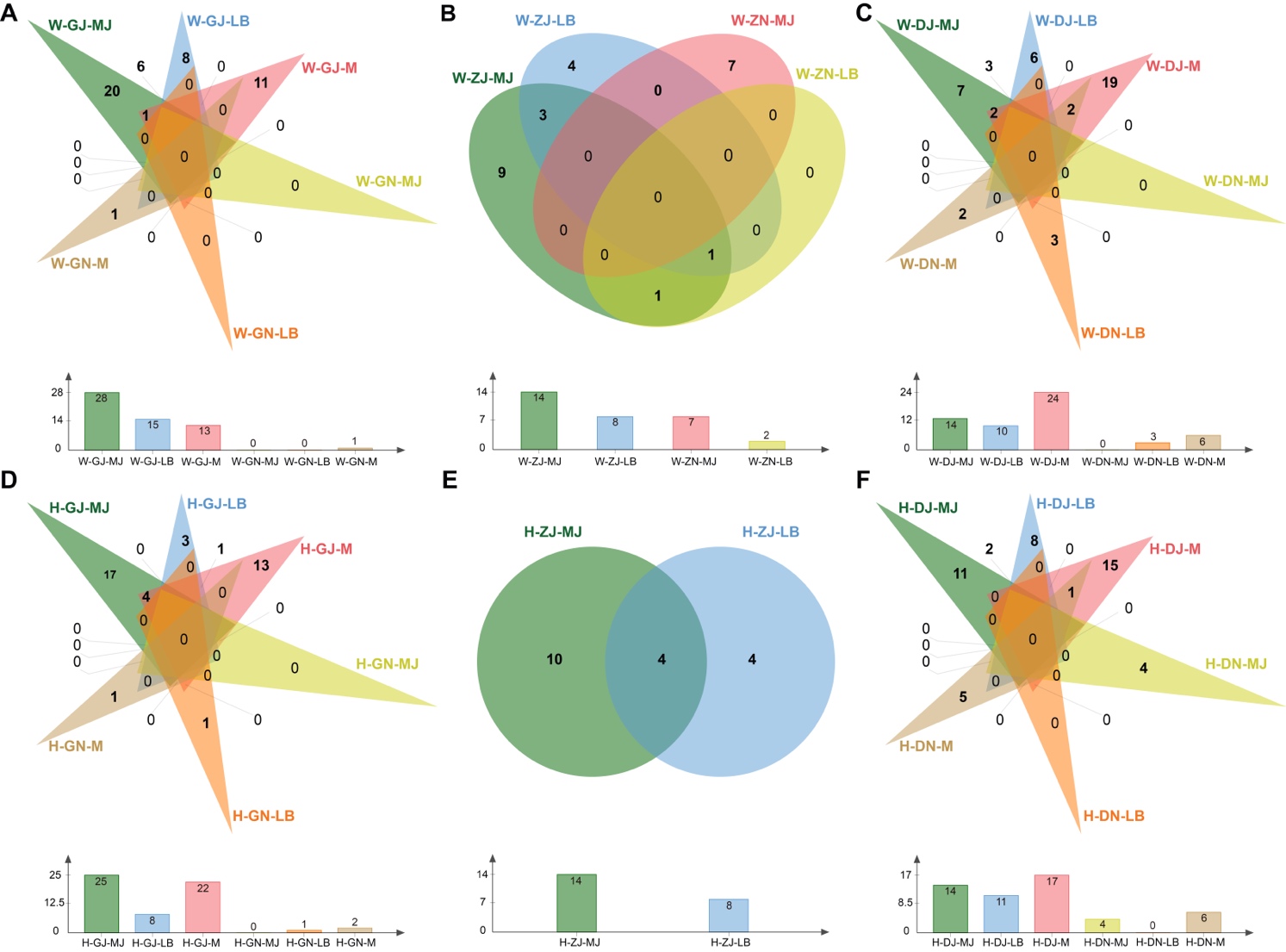


**Supplementary Figure 11.** Venn diagrams of OTU distribution for culturable bacteria across altitudes and developmental stages. **(A–C)** *G. elata* f. *glauca* from high-, middle-, and low-altitude sites, respectively (n = 8 samples). **(D–F)** *G. elata* f. *elata* from corresponding altitudes (n = 9 samples). Sample codes: Form (W, f. *glauca*; H, f. *elata*), altitude (G, high; Z, middle; D, low), habitat (J, tuberosphere soil; N, endosphere), and developmental stage (MJ, Medium *Jianma*; LB, Large *Baima*; M, *Mima*).


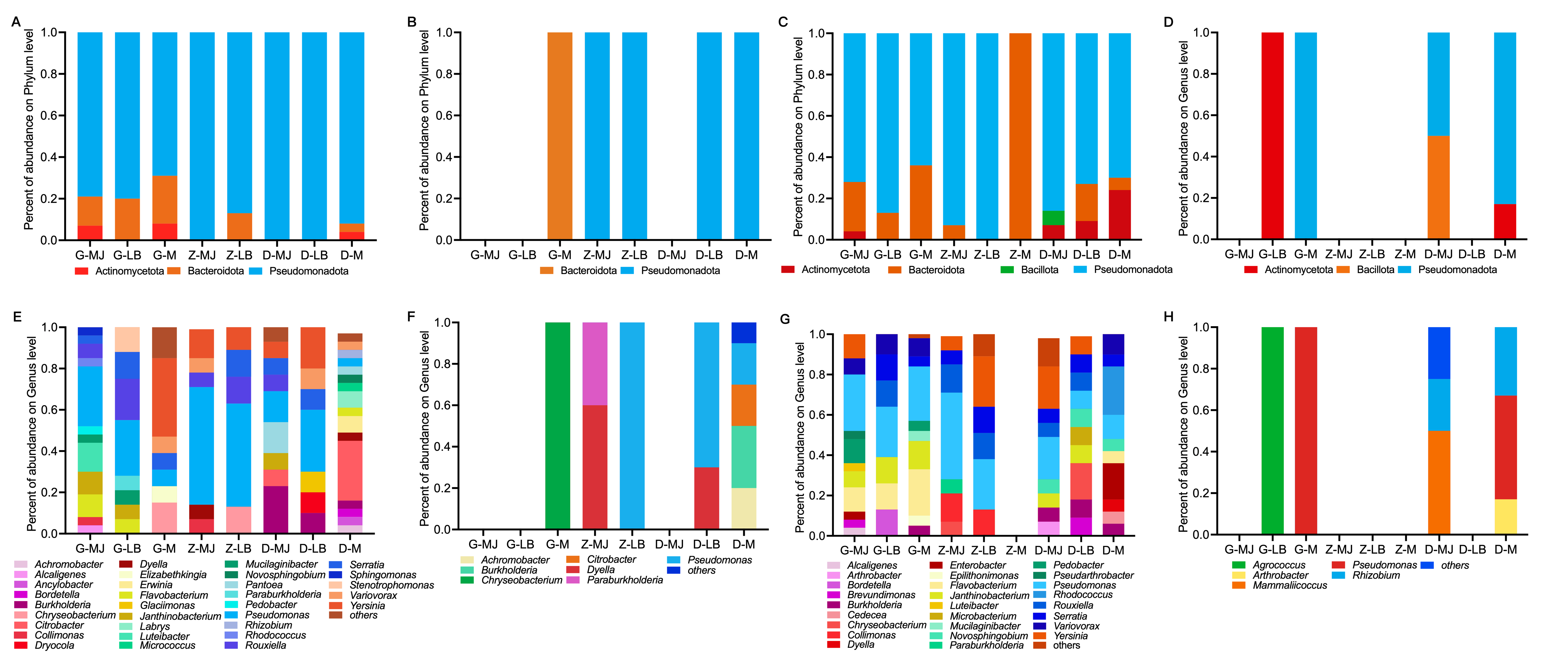


**Supplementary Figure 12.** Dynamics of culturable bacteria across developmental stages. Relative abundance of **(A–D)** bacterial phyla and **(E–H)** genera. Panels: **(A, E)** *G. elata* f. *glauca* (tuberosphere soil; n = 8 samples); **(B, F)** *G. elata* f. *glauca* (endosphere; n = 8 samples); **(C, G)** *G. elata* f. *elata* (tuberosphere soil; n = 9 samples); **(D, H)** *G. elata* f. *elata* (endosphere; n = 9 samples). Sample codes: Altitude (G, high; Z, middle; D, low) and developmental stage (MJ, Medium *Jianma*; LB, Large *Baima*; M, *Mima*).

## Supplementary Tables

**Supplementary Table 1.** List of abbreviations.

| Abbreviation | Full term | Abbreviation | Full term |
| --- | --- | --- | --- |
| ACu | Available copper | L/W ratio | Length-to-width ratio |
| AFe | Available iron | M | *Mima* |
| AK | Available potassium | MB | Medium *Baima* |
| AMn | Available manganese | MC | Total concentration of all six compounds |
| AN | Alkali-hydrolyzable nitrogen | MJ | Medium *Jianma* |
| AP | Available phosphorus | N | Endosphere |
| AT | Air temperature | NAG | β-N-acetylglucosaminidase |
| AVE | Average variance extracted | OTU | Operational taxonomic unit |
| BG | β-glucosidase | P | Relative abundance |
| CBH | β-1,4-cellobiohydrolase | PA | Parishin A |
| D | Low altitude | PB | Parishin B |
| DTPA | Diethylenetriaminepentaacetic acid | PC | Parishin C |
| E | Species evenness index | PCA | Principal component analysis |
| ECa | Exchangeable calcium | PE | Parishin E |
| EMg | Exchangeable magnesium | PLS-SEM | Partial least squares path modeling |
| Enzyme C | Sum of BG, CBH, and XS | PZ | Sum of PA, PB, PC, and PE |
| Enzyme N | Sum of NAG and LAP | RDA | Redundancy analysis |
| Enzyme P | Acid phosphatase | RH | Air relative humidity |
| G | High altitude | RSD | Relative standard deviation |
| GA | Gastrodin | S | Number of OTUs |
| GH | Sum of GA and HBA | SB | Small *Baima* |
| GLM | General linear model | SE | Standard error |
| GLMM | Generalized linear mixed models | SEC | Soil electrical conductivity |
| GOF | Goodness-of-fit | SI | Simpson’s index |
| H | *G. elata* f. *elata* | SJ | Small *Jianma* |
| **H'** | Shannon-Wiener index | SM | Soil moisture |
| HBA | *p*-hydroxybenzyl alcohol | SOC | Soil organic carbon |
| ICP-OES | Inductively coupled plasma optical emission spectrometry | ST | Soil temperature |
| ITS | Internal transcribed spacer | UPLC | Ultra-performance liquid chromatography |
| J | Tuberosphere soil | VIF | Variance inflation factor |
| LAP | Leucine aminopeptidase | W | *G. elata* f. *glauca* |
| LB | Large *Baima* | XS | β-xylosidase |
| LJ | Large *Jianma* | Z | Middle altitude |
| LMM | Linear mixed model | η²p | Partial Eta-squared |
| LSD | Least significant difference |  |  |

**Supplementary Table 2.** Site characteristics and microclimate data across the three studied altitudes in the Qinling-Daba Mountains region.

| Altitude (m) | 1953 | 1653 | 650 |
| --- | --- | --- | --- |
| Category | High | Middle | Low |
| Location | Guanmao Mountain, Jinqiao Village, Qingxi Town | Guanmao Mountain, Jinqiao Village, Qingxi Town | Fangshi Village, Zhuyuan Town |
| Coordinates | 32°22′48″ N  104°48′36″ E | 32°22′48″ N  104°48′36″ E | 32°15′0″ N  105°17′24″ E |
| Planting time | 2022/4/29 | 2022/5/1 | 2022/4/28 |
| Sampling time | 2023/3/16 | 2023/3/17 | 2023/3/15 |
| Soil texture | Powdery loam | Loam | Loam |
| ST (℃) | 8.97 ± 7.44 | 11.39 ± 7.58 | 15.5 ± 6.93 |
| AT (℃) | 8.25 ± 8.22 | 9.92 ± 8.21 | 14.55 ± 8.04 |
| SM (%) | 35.97 ± 8.49 | 27 ± 20.17 | 22.51 ± 2.59 |
| RH (%) | 81.93 ± 11.6 | 81.26 ± 12.96 | 82.07 ± 10.93 |
| SEC (μs·cm^-^¹) | 190.93 ± 30.9 | 170.41 ± 149.13 | 152.09 ± 5.02 |

Notes: Sampling was conducted within 3 consecutive days to ensure consistency across altitudes, with no significant environmental fluctuations during this period. Abbreviations are defined in **Supplementary Table 1**.

**Supplementary Table 3.** Methods and standards for soil physicochemical analyses.

| Analysis item | Measurement method / Standard | Reference / Standard code |
| --- | --- | --- |
| **Soil pH** | Potentiometric method in a 1:2.5 soil-water suspension | NY/T 1121.2-2006 |
| **Exchangeable Ca & Mg** | Ammonium acetate extraction and atomic absorption spectrometry | NY/T 1121.13-2006 |
| **Available Fe, Mn, Cu, Zn** | DTPA extraction | Lindsay and Norvell, 1978; NY/T 890-2004 |
| **Soil Texture** | Pipette method | Lu and Li, 2010; LY/T 1225-1999 |
| **Alkali-hydrolyzable N** | Alkaline hydrolysis diffusion method | LY/T 1228-2015 |
| **Available K** | Inductively coupled plasma optical emission spectrometry | DB63/T 1820-2020 |
| **Available P** |  | DB63/T 1822-2020 |
| **Soil Organic Carbon** | Potassium dichromate oxidation (Walkley-Black) with external heating | Walkley and Black, 1934; Bao, 2000 |

**Supplementary Table 4.** Information on commercial soil enzyme kits and chemical reference standards used in this study.

| Kits / Compounds | Manufacturer | Product / CAS number | Purity |
| --- | --- | --- | --- |
| β-Glucosidase | Beijing Solarbio Science & Technology Co., Ltd | BC0165 | – |
| β-1,4-Cellobiohydrolase |  | BC4035 | – |
| β-Xylosidase |  | BC4015 | – |
| β-N-Acetylglucosaminidase |  | BC4005 | – |
| Leucine Aminopeptidase |  | BC4025 | – |
| Acid Phosphatase |  | BC0145 | – |
| Gastrodin | Must (Chengdu) Biotechnology Co., Ltd. | MUST-21122205 | ≥ 98% |
| *p*-hydroxybenzyl alcohol |  | MUST-21111510 | ≥ 99% |
| Parishin A | Beijing Boye Biotech Co., Ltd. | 62499-28-9 (CAS) | ≥ 98% |
| Parishin B |  | 174972-79-3 (CAS) | ≥ 98% |
| Parishin C |  | 174972-80-6 (CAS) | ≥ 98% |
| Parishin E | Shanghai Yuanye Bio-Technology Co., Ltd. | 952068-57-4 (CAS) | ≥ 98% |

**Supplementary Table 5.** Results of the UPLC method validation for the quantification of six bioactive compounds in *G. elata*.

| Compound | GA | HBA | PA | PB | PC | PE |
| --- | --- | --- | --- | --- | --- | --- |
| Regression Equation | Y = 21177X + 250955 | Y = 44048X + 204464 | Y = 22819X + 196139 | Y = 11975X + 8433.2 | Y = 17670X - 108161 | Y = 20030X - 325296 |
| *R*² | 0.9991 | 0.9995 | 0.9992 | 0.9999 | 0.999 | 0.9993 |
| Linear range (μg·mL⁻¹) | 5-400 | 5-250 | 5-400 | 5-400 | 5-250 | 12.5-400 |
| LOD (μg·mL⁻¹) | 0.21 | 0.03 | 0.17 | 0.21 | 0.25 | 0.93 |
| LOQ (μg·mL⁻¹) | 0.63 | 0.09 | 0.52 | 0.63 | 0.75 | 2.82 |
| Stability RSD (%) | 1.96 | 0.9 | 1.49 | 2.76 | 1.4 | 0.57 |
| Precision RSD (%) | 1.01 | 0.55 | 0.75 | 1.71 | 1.74 | 0.66 |
| Repeatability RSD (%) | 1.99 | 0.71 | 0.64 | 1.35 | 2 | 1.06 |
| Avg. recovery (%) | 100.77 | 98.78 | 103.18 | 101.3 | 99.51 | 102.54 |
| Recovery RSD (%) | 0.5 | 0.72 | 0.97 | 2.25 | 1.11 | 1.15 |

Notes: The abbreviations for the compounds can be found in **Supplementary Table 1**.

**Supplementary Table 6.** Specifications of LMMs.

| Analysis | Soil enzyme activities | Tuber morphology and biomass (per stage) |
| --- | --- | --- |
| Dependent variables | Enzyme C, N, P activity | Length, Width, L/W ratio, Log (Yield), Log (Individual weight), Count |
| Fixed Effects | Altitude, Form, Developmental Stage, and all interactions | |
| Random Effect | Intercept (Cultivation Bed) | |
| Model Specification (Example) | Example (Enzyme C): Enzyme_C ~ Altitude × Form × Stage + (1 \| Bed_ID) | Example (Log (Individual Weight)): Log (Weight) ~ Altitude × Form × Stage + (1 \| Bed_ID)) |
| Distribution & Link | Gaussian, Identity | |
| Software & Procedure | SPSS (v26.0), Linear Mixed Models | |
| Post-hoc Comparisons | Šidák test | |

Note: This model structure was applied uniformly to all continuous dependent variables (e.g., other soil enzyme activities, tuber morphological and biomass traits).

**Supplementary Table 7.** Specification of the GLMM.

| Analysis | Tuber count (per stage) | |
| --- | --- | --- |
| Fixed Effects | Altitude, Form, Developmental Stage, and all interactions |  |
| Random Effect | Intercept (Cultivation Bed) |  |
| Model Specification (Example) | Log(E[Count]) ~ Alt × Form × Stage + (1 \| Bed_ID)) | |
| Distribution & Link | Negative Binomial, Log |  |
| Software & Procedure | SPSS (v26.0), Generalized Linear Mixed Model |  |
| Post-hoc Comparisons | Šidák test |  |

**Supplementary Table 8.** Specifications of the GLM models.

| Analysis | Soil properties | Tuber yield and count (per Bed) |
| --- | --- | --- |
| Dependent variables | pH, SOC, AN, AP, AK, ECa | Total tuber yield and count |
| Fixed Effects | Altitude, Form, Altitude × Form | |
| Model Specification (Example) | Example (SOC): SOC ~ Alt + Form + Altitude × Form | Example (Total Yield): Total_Yield ~ Altitude + Form + Alt × Form |
| Key Assumption Checks | 1. Homogeneity of variance (Levene’s test).  2. Normality of residuals (Q-Q plot). | |
| Software & Procedure | SPSS (v26.0), General Linear Model | |
| Post-hoc Comparisons | Šidák test | |

Note: This model structure was applied uniformly to all continuous dependent variables (e.g., other soil properties, total tuber yield and count).

**Supplementary Table 9.** Reflective measurement model specification for the PLS-SEM of *G. elata* f. *glauca*.

| Latent variable | Code |
| --- | --- |
| Microclimate | ST, Soil temperature; RH, Air relative humidity |
| Soil physicochemical properties | SOC, Soil organic carbon; AK, Available K |
| Soil enzyme activities | Enzyme C activity, Sum of BG, CBH, and XS activities; Enzyme N activity: Sum of NAG and LAP activities |
| Culturable bacterial diversity | SI, Simpson’s index; P, Yield-related bacterial relative abundance |
| Growth traits | Total tuber yield (per bed) |

Structural Model (Inner Model). Predictive paths between latent variables were defined *a priori* based on ecological theory, forming a correlative chain. Hypothesized relationships were specified in a lower-triangular binary path matrix, indicating the direction of prediction (e.g., Soil properties → Soil enzyme activities; Soil enzyme activities → Culturable bacterial diversity; all preceding constructs → Growth traits).

**Supplementary Table 10.** GLM results for the associations of altitude and *G. elata* form with bulk soil physicochemical properties (n = 3 cultivation beds).

|  | Altitude | Form | Altitude × Form |
| --- | --- | --- | --- |
| pH | *F* = 993.0 (2, 12); < 0.001^***^; η²p = 0.99 | *F* = 15.7 (1, 12); < 0.001^***^; η²p = 0.57 | *F* = 20.3 (2, 12); < 0.001^***^; η²p = 0.77 |
| SOC | *F* = 352.4 (2, 12); < 0.001^***^; η²p = 0.98 | *F* = 0.8 (1, 12); 0.15; η²p = 0.06 | *F* = 9.5 (2, 12); < 0.001^***^; η²p = 0.61 |
| AN | *F* = 720.6 (2, 12); < 0.001^***^; η²p = 0.99 | *F* = 32.4 (1, 12); < 0.001^***^; η²p = 0.73 | *F* = 8.3 (2, 12); < 0.001^***^; η²p = 0.58 |
| AP | *F* = 66.5 (2, 12); < 0.001^***^; η²p = 0.92 | *F* = 31.2 (1, 12); < 0.001^***^; η²p = 0.72 | *F* = 0.4 (2, 12); 0.203; η²p = 0.07 |
| AK | *F* = 5571.3 (2, 12); < 0.001^***^; η²p = 1.00 | *F* = 23.4 (1, 12); < 0.001^***^; η²p = 0.66 | *F* = 43.0 (2, 12); < 0.001^***^; η²p = 0.88 |
| ECa | *F* = 656.8 (2, 12); < 0.001^***^; η²p = 0.99 | *F* = 19.3 (1, 12); < 0.001^***^; η²p = 0.62 | *F* = 8.9 (2, 12); < 0.001^***^; η²p = 0.60 |

Notes: The table presents *F*-statistics with degrees of freedom (effect df, error df) in parentheses, *p*-values, and partial eta-squared (η²p) as a measure of effect size. Analyses were performed using GLMs with altitude and form as fixed factors. Each cultivation bed was considered an independent experimental unit (biological replicate). Abbreviations for soil physicochemical properties are listed in **Supplementary Table 1**. ^*^*p* < 0.05; ^**^*p* < 0.01; ^***^*p* < 0.001.

**Supplementary Table 11**. LMMs results for the associations of altitude, *G. elata* form, and developmental stage with tuberosphere soil Enzyme C, N, and P activities (n = 3 cultivation beds).

|  | Enzyme C | Enzyme N | Enzyme P |
| --- | --- | --- | --- |
| Alti. | *F* = 141.3 (2, 30); < 0.001^***^ | *F* = 35.0 (2, 30); < 0.001^***^ | *F* = 49.7 (2, 30); < 0.001^***^ |
| Form | *F* = 7.4 (1, 30); 0.011^*^ | *F* = 0.3 (1, 30); 0.613 | *F* = 5.3 (1, 30); 0.029^*^ |
| Stage | *F* = 27.6 (2, 30); < 0.001^***^ | *F* = 0.8 (2, 30); 0.439 | *F* = 1.5 (2, 30); 0.237 |
| Alti. × Form | *F* = 4.2 (2, 30); 0.024^*^ | *F* = 8.8 (2, 30); < 0.001^***^ | *F* = 3.7 (2, 30); 0.036^*^ |
| Alti. × Stage | *F* = 14.7 (4, 30); < 0.001^***^ | *F* = 16.1 (4, 30); < 0.001^***^ | *F* = 1.3 (4, 30); 0.291 |
| Form × Stage | *F* = 6.5 (1, 30); 0.016^*^ | *F* = 6.2 (1,30); 0.019^*^ | *F* = 4.9 (1, 30); 0.035^*^ |
| Alti. × Form × Stage | *F* = 2.3 (2, 30); 0.121 | *F* = 5.3 (2, 30); 0.011^*^ | *F* = 0.2 (2, 30); 0.855 |

Notes: Values are *F*-statistics with numerator and denominator degrees of freedom in parentheses, followed by *p*-values. Models included altitude, *G. elata* form, and developmental stage as fixed factors, and cultivation bed as a random intercept. Abbreviations for soil enzyme activities are listed in **Supplementary Table 1**. ^*^*p* < 0.05; ^**^*p* < 0.01; ^***^*p* < 0.001.

**Supplementary Table 12**. GLM results for the associations of altitude and *G. elata* form with total tuber yield and count per bed (n = 5 cultivation beds).

|  | Yield | Count |
| --- | --- | --- |
| Alti. | *F* = 13.6 (2, 24); < 0.001^***^; η²p = 0.53 | *F* = 4.1 (2, 24); 0.029^*^; η²p = 0.26 |
| Form | *F* = 2.0 (1, 24); 0.171; η²p = 0.08 | *F* = 10.1 (1, 24); 0.004^**^; η²p = 0.30 |
| Alti. × Form | *F* = 1.4 (2, 24); 0.273; η²p = 0.10 | *F* = 0.0 (2, 144); 0.966; η²p = 0.00 |

Notes: The table presents *F*-statistics with degrees of freedom (effect df, error df) in parentheses, *p*-values, and η²p as a measure of effect size. The analysis was performed using a GLM with altitude and form as fixed factors. Each cultivation bed was considered an independent experimental unit (biological replicate). ^*^*p* < 0.05; ^**^*p* < 0.01; ^***^*p* < 0.001.

**Supplementary Table 13**. Results of mixed-effects models testing the associations of altitude, *G. elata* form, developmental stage, and their interactions with tuber growth traits (n = 5 cultivation beds).

|  | Log (Yield) (per stage) | Count (per stage) | Length (per tuber) |
| --- | --- | --- | --- |
| Alti. | *F* = 12.0 (2, 21); < 0.001^***^ | *F* = 16.5 (2, 144); < 0.001^***^ | *F* = 14.7 (2, 34); < 0.001^***^ |
| Form | *F* = 8.0 (1, 22); 0.01^**^ | *F* = 23.3 (1, 144); < 0.001^***^ | *F* = 6.1 (1, 44); 0.018^*^ |
| Stage | *F* = 27.5 (5, 72); < 0.001^***^ | *F* = 123.5 (5, 144); < 0.001^***^ | *F* = 386.8 (5, 1343); < 0.001^***^ |
| Alti. × Form | *F* = 1.8 (2, 21); 0.192 | *F* = 14.6 (2, 144); < 0.001^***^ | *F* = 2.9 (2, 34); 0.068 |
| Alti. × Stage | *F* = 2.0 (10, 72); 0.042^*^ | *F* = 410.2 (10, 144); < 0.001^***^ | *F* = 6.3 (10, 1344); < 0.001^***^ |
| Form × Stage | *F* = 0.4 (5, 73); 0.854 | *F* = 76.1 (5, 144); < 0.001^***^ | *F* = 25.8 (5, 1343); < 0.001^***^ |
| Alti. × Form × Stage | *F* = 2.8 (9, 72); 0.007^**^ | *F* = 344.9 (10, 144); < 0.001^***^ | *F* = 14.1 (9, 1344); < 0.001^***^ |
|  | Width (per tuber) | L/W ratio (per tuber) | Log (Weight) (per tuber) |
| Alti. | *F* = 26.9 (2, 31); < 0.001^***^ | *F* = 16.4 (2, 36); < 0.001^***^ | *F* = 12.6 (2, 31); < 0.001^***^ |
| Form | *F* = 15.8 (1, 40); < 0.001^***^ | *F* = 0.2 (1, 53); 0.645 | *F* = 0.4 (1, 39); 0.546 |
| Stage | *F* = 530.6 (5, 1343); < 0.001^***^ | *F* = 8.0 (5, 1344); < 0.001^***^ | *F* = 948.0 (5, 1342); < 0.001^***^ |
| Alti. × Form | *F* = 6.6 (2, 31); 0.004^**^ | *F* = 5.1 (2, 37); 0.011^*^ | *F* = 2.8 (2, 32); 0.079 |
| Alti. × Stage | *F* = 8.7 (10, 1343); < 0.001^***^ | *F* = 10.0 (10, 1344); < 0.001^***^ | *F* = 3.6 (10, 1342); < 0.001^***^ |
| Form × Stage | *F* = 8.9 (5, 1343); < 0.001^***^ | *F* = 8.9 (5, 1344); < 0.001^***^ | *F* = 9.6 (5, 1342); < 0.001^***^ |
| Alti. × Form × Stage | *F* = 2.6 (9, 1343); 0.006^**^ | *F* = 6.6 (9, 1343); < 0.001^***^ | *F* = 6.2 (9, 1342); < 0.001^***^ |

Notes: Values are *F*-statistics with numerator and denominator degrees of freedom in parentheses, followed by *p*-values. Models included altitude, *G. elata* form, and developmental stage as fixed factors, and cultivation bed as a random intercept. Tuber count was analyzed using a GLMM (Poisson distribution); all other traits were analyzed using LMMs. ^*^*p* < 0.05; ^**^*p* < 0.01; ^***^*p* < 0.001.

**Supplementary Table 14.** Concentration of bioactive compounds in tubers of *G. elata* f. *glauca* across developmental stages and altitudes (n = 3 per altitude-stage group).

| Comp.  (mg·g⁻¹) | Alti. | LJ | MJ | SJ | LB | MB | SB |
| --- | --- | --- | --- | --- | --- | --- | --- |
| GA | G | 2.42 ± 0.04 | 2.3 ± 0.04 | 1.85 ± 0.06 | 3.68 ± 0.09 | 2.89 ± 0.08 | 2.84 ± 0.03 |
|  | Z | – | 8.06 ± 0.09 | 6.02 ± 0.07 | 13.88 ± 0.18 | 6.2 ± 0.17 | 5.64 ± 0.01 |
|  | D | 2.45 ± 0.07 | 4.93 ± 0.22 | 5.45 ± 0.03 | 4.13 ± 0.11 | 3.84 ± 0.06 | 4.14 ± 0.06 |
| HBA | G | 0.74 ± 0.01 | 0.92 ± 0.02 | 0.68 ± 0.02 | 1.54 ± 0.04 | 1.48 ± 0.04 | 1.81 ± 0.02 |
|  | Z | – | 0.34 ± 0 | 0.47 ± 0 | 0.31 ± 0.01 | 1.64 ± 0.02 | 2.46 ± 0 |
|  | D | 0.57 ± 0.01 | 0.22 ± 0.02 | 1.04 ± 0.01 | 1.53 ± 0.04 | 2 ± 0.01 | 2.98 ± 0.05 |
| GH | G | 3.16 ± 0.03 | 3.22 ± 0.03 | 2.53 ± 0.07 | 5.22 ± 0.13 | 4.38 ± 0.11 | 4.65 ± 0.04 |
|  | Z | – | 8.4 ± 0.09 | 6.49 ± 0.08 | 14.19 ± 0.21 | 7.85 ± 0.21 | 8.1 ± 0.01 |
|  | D | 3.03 ± 0.08 | 5.15 ± 0.24 | 6.49 ± 0.04 | 5.66 ± 0.15 | 5.84 ± 0.07 | 7.12 ± 0.11 |
| PA | G | 3.31 ± 0.01 | 4.35 ± 0.07 | 5.31 ± 0.07 | 5.71 ± 0.14 | 5.37 ± 0.11 | 6.71 ± 0.06 |
|  | Z | – | 16.61 ± 0.1 | 15.84 ± 0.09 | 18.59 ± 0.34 | 12.62 ± 0.31 | 14.39 ± 0 |
|  | D | 4.61 ± 0.07 | 14.01 ± 0.32 | 13.57 ± 0.07 | 12.27 ± 0.24 | 10.11 ± 0.08 | 11.92 ± 0.14 |
| PB | G | 5.79 ± 0.02 | 5.48 ± 0.1 | 5.56 ± 0.2 | 7.38 ± 0.14 | 6.1 ± 0.12 | 7.4 ± 0.10 |
|  | Z | – | 12.7 ± 0.12 | 11.35 ± 0.07 | 13.11 ± 0.26 | 11.32 ± 0.21 | 12.36 ± 0.2 |
|  | D | 6.16 ± 0.1 | 10.73 ± 0.23 | 10.44 ± 0.05 | 11.11 ± 0.2 | 9.31 ± 0.12 | 10.17 ± 0.12 |
| PC | G | 1.33 ± 0.01 | 1.23 ± 0.01 | 1.03 ± 0.03 | 1.43 ± 0.02 | 1.25 ± 0.02 | 1.43 ± 0.02 |
|  | Z | – | 2.31 ± 0.02 | 1.96 ± 0.03 | 3.05 ± 0.07 | 2 ± 0.04 | 2.48 ± 0 |
|  | D | 1.57 ± 0.01 | 2.1 ± 0.05 | 1.96 ± 0.02 | 1.93 ± 0.01 | 1.37 ± 0.02 | 1.62 ± 0.02 |
| PE | G | 10.65 ± 0.04 | 10.76 ± 0.15 | 10.3 ± 0.23 | 11.45 ± 0.22 | 11.67 ± 0.23 | 11.42 ± 0.08 |
|  | Z | – | 16.54 ± 0.14 | 17.04 ± 0.23 | 14.35 ± 0.39 | 13.28 ± 0.27 | 14.08 ± 0.01 |
|  | D | 5.86 ± 0.1 | 15.27 ± 0.42 | 15.74 ± 0.12 | 8.75 ± 0.16 | 14.43 ± 0.14 | 11.65 ± 0.12 |
| PZ | G | 21.08 ± 0.07 | 21.82 ± 0.33 | 22.2 ± 0.45 | 25.97 ± 0.48 | 24.38 ± 0.49 | 26.96 ± 0.23 |
|  | Z | – | 48.16 ± 0.36 | 46.19 ± 0.37 | 49.1 ± 1.05 | 39.22 ± 0.81 | 43.31 ± 0.21 |
|  | D | 18.21 ± 0.24 | 42.12 ± 1.02 | 41.72 ± 0.26 | 34.06 ± 0.61 | 35.23 ± 0.35 | 35.36 ± 0.39 |
| MC | G | 48.48 ± 0.14 | 50.08 ± 0.68 | 49.46 ± 0.88 | 62.38 ± 1.2 | 57.52 ± 1.18 | 63.22 ± 0.52 |
|  | Z | – | 113.13 ± 0.84 | 105.37 ± 0.9 | 126.59 ± 2.52 | 94.14 ± 2.02 | 102.82 ± 0.44 |
|  | D | 42.46 ± 0.64 | 94.53 ± 2.51 | 96.43 ± 0.58 | 79.43 ± 1.52 | 82.12 ± 0.79 | 84.97 ± 1 |

Notes: Data are presented as mean ± standard deviation. G, High; Z, Middle; D, Low. Abbreviations for compounds and developmental stages are listed in **Supplementary Table 1**. The symbol “–” indicates missing data for the *f. glauca* Large *Jianma* (LJ) stage at middle altitude due to sample limitations.

**Supplementary Table 15.** Concentration of bioactive compounds in tubers of *G. elata* f. *elata* across developmental stages and altitudes (n = 3 per altitude-stage group).

| stage | Altitude | GA (mg·g⁻¹) | HBA (mg·g⁻¹) | GH (mg·g⁻¹) | PA (mg·g⁻¹) | PB (mg·g⁻¹) | PC (mg·g⁻¹) | PE (mg·g⁻¹) | PZ (mg·g⁻¹) | MC (mg·g⁻¹) |
| --- | --- | --- | --- | --- | --- | --- | --- | --- | --- | --- |
| LJ | High | 3.83 ± 0.12 | 0.37 ± 0.01 | 4.19 ± 0.12 | 10.17 ± 0.06 | 9.76 ± 0.05 | 2.33 ± 0.02 | 5.63 ± 0.02 | 27.89 ± 0.14 | 64.16 ± 0.47 |
|  | Middle | 6.77 ± 0.09 | 0.12 ± 0.01 | 6.89 ± 0.09 | 13.22 ± 0.08 | 11.62 ± 0.08 | 3.3 ± 0.03 | 6.03 ± 0.05 | 34.17 ± 0.23 | 82.12 ± 0.65 |
|  | Low | 4.4 ± 0.05 | ND | 4.4 ± 0.05 | 11.44 ± 0.1 | 9.18 ± 0.08 | 2.22 ± 0.04 | 6.95 ± 0.06 | 29.79 ± 0.27 | 68.39 ± 0.62 |
| MJ | High | 2.96 ± 0.07 | 0.92 ± 0.01 | 3.88 ± 0.08 | 12.67 ± 0.09 | 9.33 ± 0.05 | 1.92 ± 0.01 | 5.54 ± 0.03 | 29.47 ± 0.18 | 66.69 ± 0.51 |
|  | Middle | 4.35 ± 0.03 | 0.14 ± 0.01 | 4.49 ± 0.03 | 15.28 ± 0.04 | 10.33 ± 0.05 | 2.53 ± 0.01 | 6.66 ± 0.04 | 34.8 ± 0.12 | 78.56 ± 0.3 |
|  | Low | 4.56 ± 0.04 | 0.12 ± 0.01 | 4.68 ± 0.04 | 13.97 ± 0.06 | 9.65 ± 0.07 | 1.85 ± 0.01 | 7.33 ± 0.06 | 32.8 ± 0.19 | 74.97 ± 0.44 |
| SJ | High | 2.75 ± 0.04 | 1.03 ± 0.02 | 3.78 ± 0.04 | 11.83 ± 0.09 | 9.04 ± 0.1 | 1.82 ± 0.02 | 5.75 ± 0.04 | 28.44 ± 0.24 | 64.43 ± 0.55 |
|  | Middle | 4.41 ± 0.07 | 0.2 ± 0.01 | 4.61 ± 0.08 | 15.49 ± 0.05 | 10.38 ± 0.06 | 2.15 ± 0.02 | 6.88 ± 0.03 | 34.9 ± 0.12 | 79.01 ± 0.37 |
|  | Low | 3.48 ± 0.24 | 0.02 ± 0.01 | 3.5 ± 0.25 | 14.48 ± 0.42 | 9.51 ± 0.24 | 1.84 ± 0 | 7.93 ± 0.36 | 33.76 ± 1.02 | 74.52 ± 2.52 |
| LB | High | 4.9 ± 0.09 | 0.94 ± 0.02 | 5.84 ± 0.1 | 11.13 ± 0.19 | 10.26 ± 0.15 | 1.91 ± 0.03 | 6.79 ± 0.11 | 30.08 ± 0.48 | 71.83 ± 1.15 |
|  | Middle | 4.17 ± 0.05 | 1.53 ± 0.01 | 5.69 ± 0.06 | 12.4 ± 0.13 | 10.41 ± 0.21 | 1.8 ± 0.02 | 6.47 ± 0.05 | 31.09 ± 0.36 | 73.56 ± 0.84 |
|  | Low | 3.45 ± 0.01 | 1.93 ± 0.05 | 5.38 ± 0.06 | 10.29 ± 0.05 | 10.43 ± 0.03 | 2.26 ± 0.02 | 6.76 ± 0.03 | 29.73 ± 0.11 | 70.22 ± 0.32 |
| MB | High | 4.94 ± 0.11 | 1.61 ± 0.02 | 6.55 ± 0.13 | 12.39 ± 0.14 | 10.74 ± 0.16 | 2.03 ± 0.03 | 8.51 ± 0.14 | 33.67 ± 0.47 | 80.44 ± 1.19 |
|  | Middle | 6.93 ± 0.11 | 0.89 ± 0.03 | 7.82 ± 0.14 | 16.96 ± 0.07 | 11.7 ± 0.04 | 2.78 ± 0.03 | 9.15 ± 0.05 | 40.58 ± 0.17 | 96.8 ± 0.62 |
|  | Low | 6.4 ± 0.16 | 1.41 ± 0.01 | 7.8 ± 0.17 | 15.84 ± 0.11 | 13.3 ± 0.12 | 3.31 ± 0.13 | 8.78 ± 0.08 | 41.24 ± 0.31 | 98.08 ± 0.86 |
| SB | High | 2.01 ± 0.05 | 4.19 ± 0.05 | 6.2 ± 0.09 | 6.08 ± 0.08 | 7.45 ± 0.09 | 1.28 ± 0.12 | 5.8 ± 0.06 | 20.62 ± 0.34 | 53.63 ± 0.84 |
|  | Middle | 9.08 ± 0.11 | 3.98 ± 0.06 | 13.06 ± 0.16 | 13.92 ± 0.09 | 11.5 ± 0.33 | 2.88 ± 0.17 | 9.22 ± 0.24 | 37.52 ± 0.44 | 101.16 ± 1.16 |
|  | Low | 4.85 ± 0.02 | 2.63 ± 0.03 | 7.48 ± 0.05 | 14.25 ± 0.09 | 12.82 ± 0.03 | 3.26 ± 0.01 | 7.7 ± 0.02 | 38.03 ± 0.11 | 91.01 ± 0.31 |
| M | High | 7.19 ± 0.01 | 2.52 ± 0.01 | 9.71 ± 0.02 | 16.17 ± 0.05 | 12.77 ± 0.11 | 2.74 ± 0.2 | 8.56 ± 0.03 | 40.24 ± 0.21 | 99.91 ± 0.39 |
|  | Middle | 9.6 ± 0.04 | 0.46 ± 0.01 | 10.06 ± 0.04 | 20.64 ± 0.07 | 11.99 ± 0.11 | 3.46 ± 0.02 | 8.22 ± 0.03 | 44.31 ± 0.16 | 108.73 ± 0.34 |
|  | Low | 6.78 ± 0.39 | 2.42 ± 0.12 | 9.2 ± 0.51 | 15.36 ± 0.44 | 13.59 ± 0.35 | 3.46 ± 0.06 | 8.89 ± 0.36 | 41.3 ± 1.19 | 101.01 ± 3.38 |

Notes: Data are presented as mean ± standard deviation. Abbreviations for compounds and developmental stages are listed in **Supplementary Table 1**. ND, not detected.

Supplementary Table 16. Molecular identification of culturable fungal isolates from tubers of *G. elata* f. *glauca* and *G. elata* f*. elata*.

| Strain ID | Altitude | Form | Stage | Habitat | GenBank accession | Taxa | Similarity (%) |
| --- | --- | --- | --- | --- | --- | --- | --- |
| F1 | High | f. *glauca* | LB | Tuberosphere | MH532834.1 | *Cordyceps fumosorosea* | 100 |
| F2 | High | f. *glauca* | LB | Tuberosphere | MN817709.1 | *Ilyonectria mors-panacis* | 100 |
| F3 | High | f. *glauca* | M | Endosphere | MH790219.1 | *Aspergillus versicolor* | 100 |
| F4 | High | f. *elata* | MJ | Endosphere | MT133754.1 | *Sistotrema brinkmannii* | 100 |
| F5 | High | f. *elata* | MJ | Endosphere | MN602591.1 | *Metarhizium rileyi* | 99.83 |
| F6 | High | f. *elata* | LB | Endosphere | MH790219.1 | *Aspergillus versicolor* | 100 |
| F7 | Middle | f. *elata* | LB | Endosphere | OP039318.1 | *Coprinellus radians* | 99.84 |

Notes: F3 and F6 share the same GenBank accession due to 100% sequence similarity. No culturable fungal isolates were obtained from the remaining altitude-stage groups.
